# Structural and molecular insight into antibody recognition of dynamic neoepitopes in membrane tethered MUC1 of pancreatic cancer cells and secreted exosomes†

**DOI:** 10.1039/d3cb00036b

**Published:** 2023-05-24

**Authors:** Hajime Wakui, Yasuhiro Yokoi, Chieko Horidome, Toyoyuki Ose, Min Yao, Yoshikazu Tanaka, Hiroshi Hinou, Shin-Ichiro Nishimura

**Affiliations:** a Field of Drug Discovery Research, Faculty of Advanced Life Science, and Graduate School of Life Science, Hokkaido University N21 W11 Kita-ku Sapporo 001-0021 Japan shin@sci.hokudai.ac.jp; b Field of X-ray Structural Biology, Faculty of Advanced Life Science, and Graduate School of Life Science, Hokkaido University N10 W8 Kita-ku Sapporo 060-0810 Japan; c Graduate School of Life Sciences, Tohoku University 2-1-1 Katahira Aoba-ku Sendai 980-8577 Japan

## Abstract

Pancreatic cancer is highly metastatic and has poor prognosis, mainly due to delayed detection, often after metastasis has occurred. A novel method to enable early detection and disease intervention is strongly needed. Here we unveil for the first time that pancreatic cancer cells (PANC-1) and secreted exosomes express MUC1 bearing cancer-relevant dynamic epitopes recognized specifically by an anti-MUC1 antibody (SN-131), which binds specifically core 1 but not core 2 type *O*-glycans found in normal cells. Comprehensive assessment of the essential epitope for SN-131 indicates that PANC-1 cells produce dominantly MUC1 with aberrant *O*-glycoforms such as Tn, T, and sialyl T (ST) antigens. Importantly, SN-131 showed the highest affinity with MUC1 bearing ST antigen at the immunodominant DTR motif (*K*_D_ = 1.58 nM) independent of the glycosylation states of other Ser/Thr residues in the MUC1 tandem repeats. The X-ray structure revealed that SN-131 interacts directly with Neu5Ac and root GalNAc of the ST antigen in addition to the proximal peptide region. Our results demonstrate that targeting *O*-glycosylated “dynamic neoepitopes” found in the membrane-tethered MUC1 is a promising therapeutic strategy for improving the treatment outcome of patients with pancreatic cancer.

## Abstract 2

We unveil for the first time that pancreatic cancer cells (PANC-1) and secreted exosomes express MUC1 bearing cancer-relevant dynamic epitopes recognized specifically by an anti-MUC1 antibody (SN-131).

## Graphical abstract

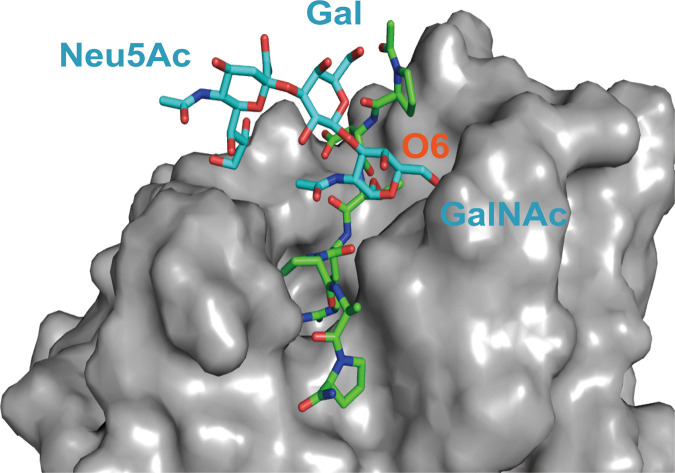

## Introduction

One of the most lethal cancers, pancreatic cancer, has a 5-year survival rate of less than 5% due to difficulties in detecting early-stage disease, its high metastatic potential, and resistance to existing conventional therapies.^1^ Despite the introduction of new therapeutic regimens, the outcome has not markedly improved over the past few decades.^2^ As opposed to other malignant cancers, immune checkpoint inhibitory antibodies targeting cytotoxic T lymphocyte assisted protein 4 (CTLA-4), programmed cell death 1 (PD-1), or programmed death ligand 1 (PD-L1) were shown to have no clinical benefit in patients suffering with pancreatic cancer.^3,4^ Therefore, novel approaches to more effective treatment of advanced and metastatic pancreatic cancer are urgently required.

Structural alteration in protein glycosylation is well known to be a general characteristic feature of malignancy and often results in up-regulated sialylation.^5,6^ Given that interaction of members of the Siglec family expressed by various immune cells with sialic acid is an alternative mechanism for cancer immune evasion to the immune checkpoint pathway,^7–11^ cancer specific membrane glycoproteins carrying sialylated glycans may be highly potential target molecules for the development of novel diagnostic and therapeutic reagents.

**Fig. 1:**
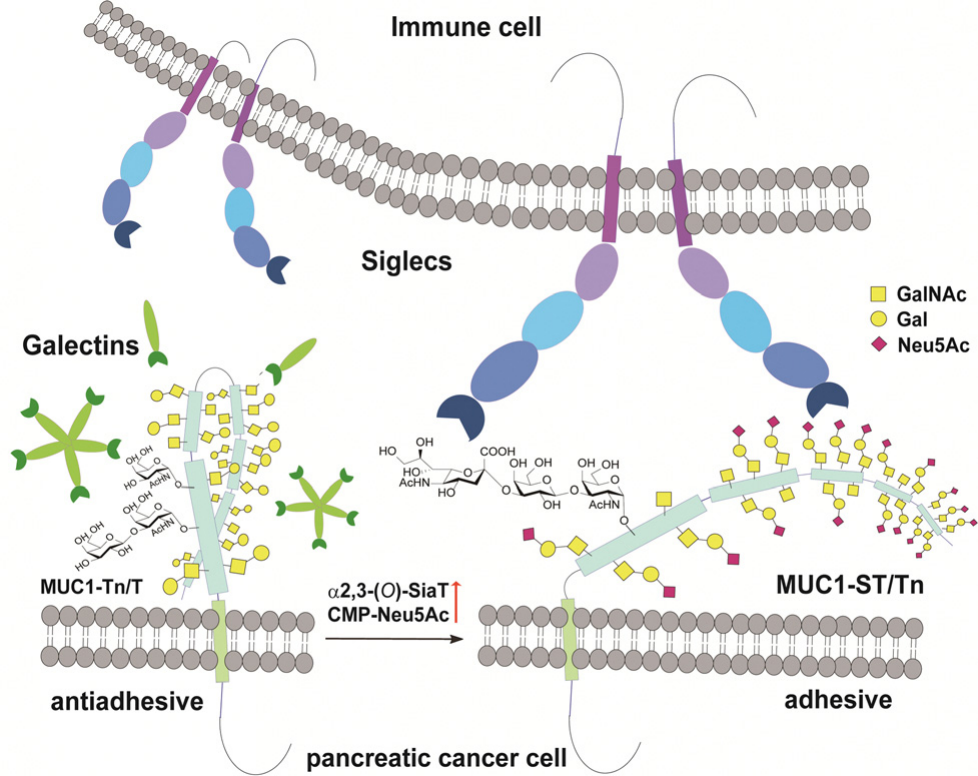
Interaction between pancreatic cancer cell surface MUC1 and immune cell surface Siglecs depends strongly on *O*-glycosylation states in the MUC1 TRDs. Siglecs of immune cells such as Siglec-7 and Siglec-9 bind preferentially to the adhesive tumour cell surface MUC1 TRD bearing multiple ST (Neu5Acα2,3Galβ1,3GalNAcα) antigens, while antiadhesive tumour cell surface MUC1 TRD bearing asialo-*O*-glycans such as Tn (GalNAcα) and T (Galβ1,4GalNAcα) antigens may be recognized dominantly with Galectins mainly, Galectin-3.

MUC1 is ranked the second best potential target out of 75 tumour-associated antigens by the Translational Research Working Group of a pilot study conducted by the National Cancer Institute (NCI).^12^ MUC1, a type 1 single-pass transmembrane mucin glycoprotein highly expressed widely in adenocarcinomas including pancreatic cancer,^13–15^ modulates cell–cell interactions by altering the *O*-glycosylation profiles in its tandem repeating domain (TRD) composed of 20 amino acid residues.^16,17^ It is well known that cancer cells express a high level of MUC1 tandem repeating domain (TRD) modified with immature *O*-glycans such as Tn (GalNAcα), sialyl Tn (STn, Neu5Acα2,6GalNAcα), T (Galβ1,3GalNAcα), and sialyl T (ST, Neu5Acα2,3Galβ1,3GalNAcα1) antigens.^6,18^ Accumulated results provide evidence that interaction of MUC1 TRD with these aberrant *O*-glycoforms attached to an immunodominant DTR motif with Galectins and Siglecs is a critical mechanism for promoting cancer proliferation and metastasis, in which alteration of the glycan structures defines functional roles of MUC1 TRD in cancer biology (Fig. 1).^5,7^

Emerging importance of MUC1 TRD in cancer progression has prompted us to create antibodies recognizing MUC1 TRD bearing cancer-specific Tn, STn, T, and ST antigens.^19,20^ In particular, our attention was directed to the effect of *O*-glycosylation status, occupancy and glycoform at five potential sites within TRD, on the antibody recognition in terms of the binding specificity and affinity strength with glycopeptidic epitopes.^21–24^ The present study reveals structural and molecular basis in the specific interaction of anti-MUC1 mAb (SN-131) with “dynamic neoepitope” in the membrane-tethered MUC1 TRDs of the pancreatic cells and secreted exosomes.

## Results

### Anti-MUC1 antibody (SN-131) discriminates between MUC1 TRDs with core 1 and core 2-type *O*-glycans

**Fig. 2:**
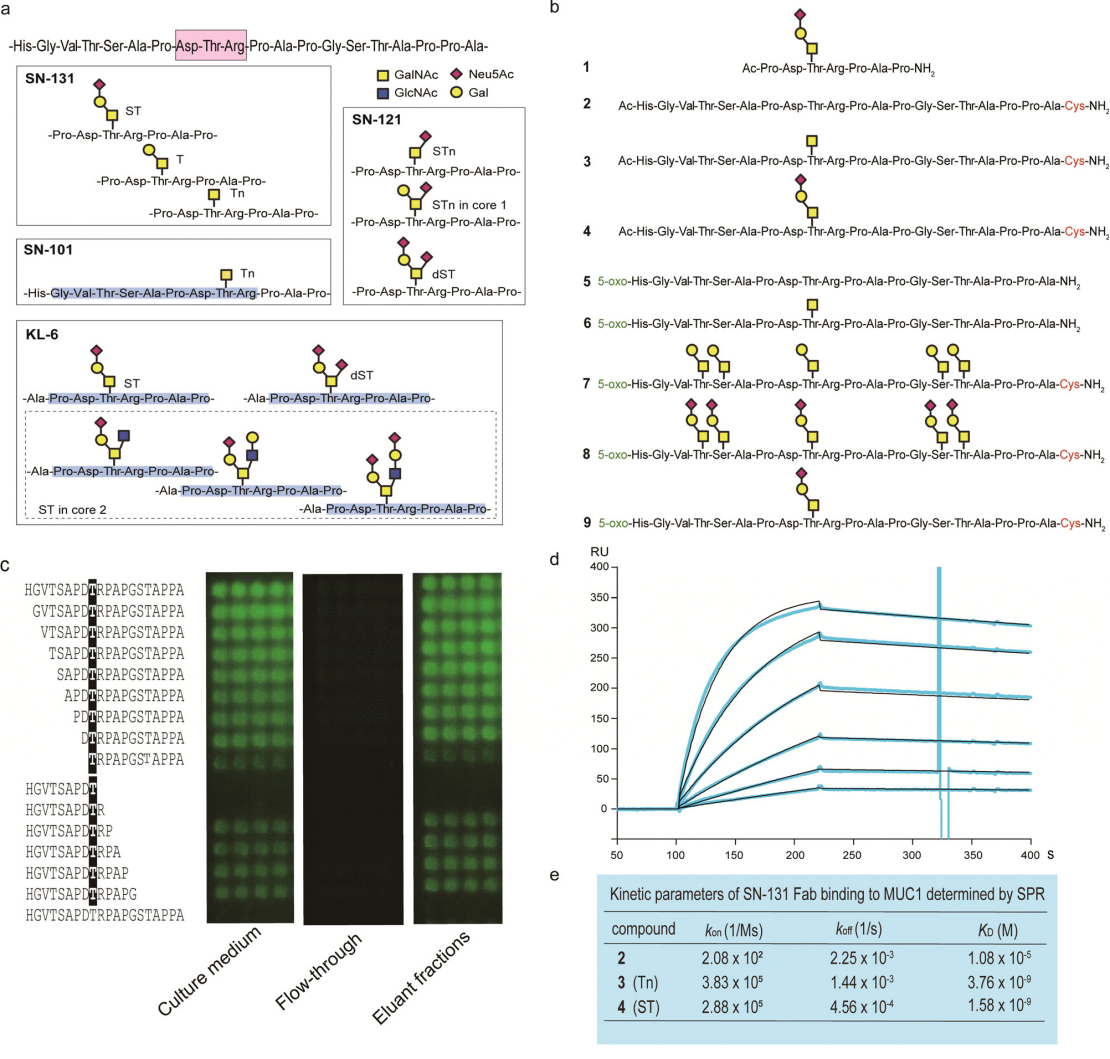
Synthetic glycopeptide library enables creation and precise characterization of epitope-defined monoclonal antibodies targeting dynamic epitopes. (a) Dynamic epitopes recognized by SN-131 and biological functions are still unclear while SN-101 and KL-6 antibodies have been fully characterized.^19–24^ (b) A list of the compounds 1–9 used in this study, in which *N*- and *C*-terminals were designed suitably for the protocols needed for purposes such as ELISA, SPR, microarray, nanosomes, and X-ray crystallography of the antibody-antigen cocrystals. 5-Oxo; 5-oxohexanoic acid. (c) Epitope mapping analyses using microarray were employed for monitoring and evaluating the activity/stability of the SN-131 antibody. SN-131 did not bind to non-glycosylated MUC1 TRD (bottom lane) and glycopeptides lacking immunodominant amino acids involved in the dynamic epitope. (d) SPR analysis of the interaction of SN-131 Fab with MUC1-ST (4) immobilized. (e) Kinetic parameters in the interaction of SN-131 Fab with MUC1 TRD 2–4 determined by SPR.

By using a robust synthetic glycopeptide library,^21–24^ we have established a straightforward approach to antibodies recognizing cancer-relevant glycopeptidic epitopes modified with aberrant *O*-glycans, namely “dynamic neoepitopes”, found in the MUC1 TRD (Fig. 2a).^19,20^ SN-131 (clone 1B2, IgG2a) is one of the anti-MUC1 mAbs created on the basis of this strategy that binds MUC1 TRD with Tn, T, and ST antigens attached to the immunodominant DTR motif (Fig. S1, ESI†). In contrast, SN-121 (clone 12D10, IgG1) reacts dominantly with this peptide region containing a STn antigen,^19^ while SN-101 (IgG1) binds MUC1 TRD only when the threonine residue is modified with the simplest non-sialylated Tn antigen.^20^ We have also demonstrated that anti-MUC1/KL6 mAb established by Kohno *et al.*,^25^ an antibody probing MUC1 TRD as a sensitive human serum biomarker of interstitial lung diseases,^26^ binds specifically to the heptapeptide moiety, Pro-Asp-Thr-Arg-Pro-Ala-Pro, bearing some sialylated *O*-glycans containing ST antigen, not only core 1-type structures but also a disialyl T [Neu5Acα2,3Galβ1,3(Neu5Acα2,6)GalNAcα] structure (dST) and even core 2-type structure, Neu5Acα2,3Galβ1,3(Neu5Acα2,3Galβ1,4GlcNAcβ1,6)GalNAcα, found commonly in normal cell surfaces.^21–23^

It is important to note that SN-131 does not bind to MUC1 TRD when the threonine residue of this immunodominant region is modified with STn, dST, or core 2-type mature complex *O*-glycan structures (Fig. 2a).^19^ We considered that SN-131 is a highly potential anti-MUC1 antibody because this antibody binds specifically MUC1 TRD having core 1 type *O*-glycans overexpressed in many cancers including pancreatic cancer but not core 2 type *O*-glycans found in normal cells. However, the structural and molecular basis of the epitope recognition by SN-131 remain to be fully understood. We needed to investigate entirely the molecular mechanism in the interaction of SN-131 with its dynamic epitope within the MUC1 TRD. Particularly, the specificity and affinity strength in the binding to dynamic epitopes should be precisely characterized in addition to the three-dimensional (3D) structure of the antibody-epitope complex. To conduct a comprehensive analyses of antibody recognition, we synthesized glycopeptide derivatives 1–9 based on a strategy by combining microwave-assisted solid-phase synthesis and enzymatic sugar extension (Fig. 2b and Scheme S1, ESI†).^21–24^ Epitope mapping and SPR analysis demonstrated that SN-131 exhibits high affinities (K_D_ = 3.78 nM and 1.58 nM) with MUC1 glycopeptide bearing Tn and ST antigens at the Asp-Thr-Arg-Pro moiety (3) and (4) (Fig. 2d, e and Fig. S2, ESI†). Notably, SN-131 binds compounds 7–9 in a similar manner, demonstrating that modification status at four other *O*-glycosylation sites does not affect the binding of SN-131 to the dynamic epitope at an immunodominant region (Fig. S3, ESI†). Despite extensive efforts for the development of many anti-MUC1 mAbs as potential candidates of anticancer therapeutic reagents for over 30 years,^27^ to our knowledge, SN-131 is the only antibody that exhibits specific and strong binding affinity with cancer-relevant MUC1 TRD modified by Tn and core 1 type *O*-glycans such as T and ST antigens at the Asp-Thr-Arg motif. Merit of antibody interacting simultaneously with MUC1 TRD having cancer-specific Tn and core 1 type *O*-glycans including T and ST antigens is clear because human normal cells have been known to express MUC1 modified dominantly with mature and complicated core 2 type *O*-glycans.^15,28^

### Crystal structures of SN-131 and its complex with ST-MUC1 glycopeptide

Given the emerging importance of the roles of MUC1 TRD-STs in the complex immune systems through the interaction with members of the Siglec family,^7,11,29,30^ our interest was centred on the structural basis in the interaction of SN-131 with MUC1 TRD-ST(s) and the potentials of SN-131 as a new class of anticancer therapeutic reagents targeting the pancreatic cancer cells. Importantly, understanding of the precise 3D structure and molecular details in antibody recognition of the dynamic epitope is indispensable for preventing an off-targeting effect by the antibody-based anticancer therapy.

**Fig. 3:**
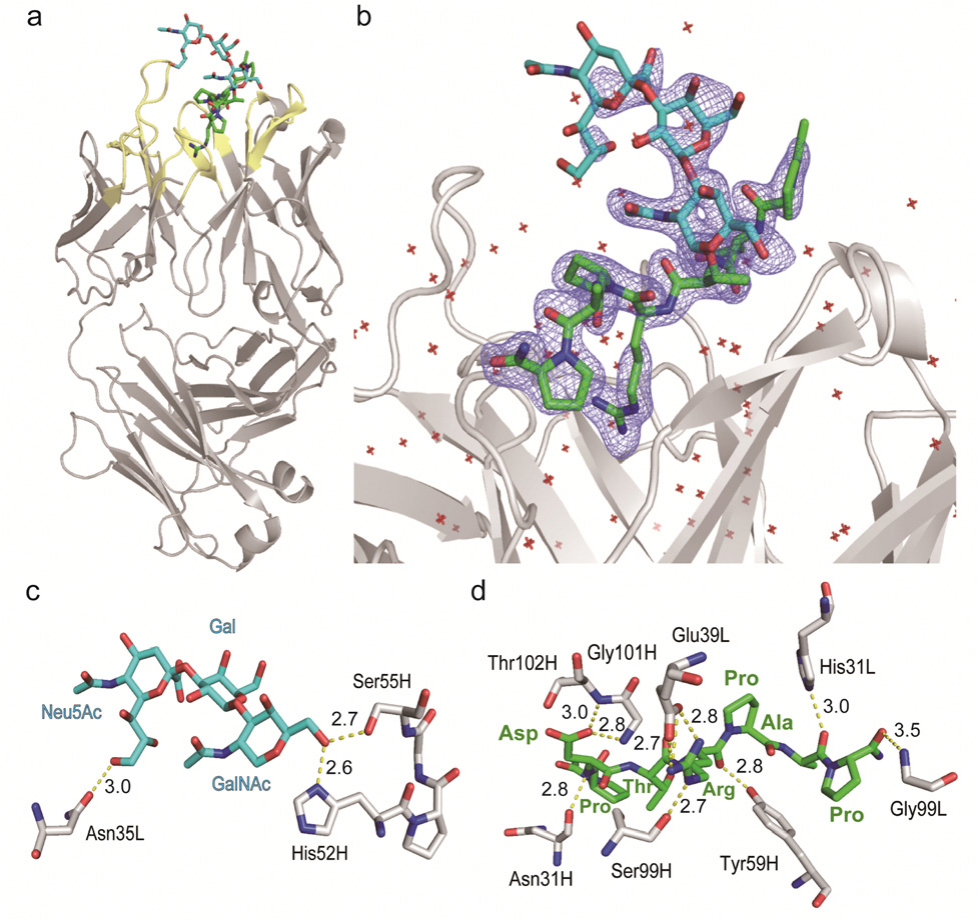
Crystal structure of SN-131 Fab in complex with MUC1 glycopeptide (1) (PDB: 8HRH). (a) Overall structure and an enlarged picture focusing on the combining site of SN-131 Fab complexed with dynamic epitope 1 represented by (2Fo–Fc) electron density map (b). (c) 3D structure focusing on glycan recognition by SN-131. Binding interaction with the Neu5Ac residue is mediated by hydrogen bonding between *O*-9 of Neu5Ac and Asn35 of the light chain, while the interaction with the GalNAc residue is mediated by hydrogen bonds between *O*-6 of GalNAc with His52 and Ser55 of the light chain. (d) 3D structure focusing on peptide recognition by SN-131. Binding of the immunodominant Pro-Asp-Thr-Arg-Pro-Ala-Pro region is mediated by multiple hydrogen bonds, salt bridges, and CH/p interactions with 8 amino acid residues in the light and heavy chains. Distances (Å) of the hydrogen bonds are represented.

To decipher the structural basis in the recognition of MUC1 TRD-ST by SN-131 antibody at the atomic level, a pure Fab fragment of SN-131 was crystalized in the presence of synthetic MUC1 TRD glycopeptide 1, a heptapeptide derivative Ac-Pro-Asp-Thr(ST)-Arg-Pro-Ala-Pro-NH_2_. The crystal of SN-131 Fab complexed with MUC1 TRD glycopeptide 1 enabled structural analysis at 2.06 Å resolution (Table S1, ESI†). The overall structure of the complex showed that the MUC1 glycopeptide 1 is bound in the antigen binding site of SN-131 Fab, which is composed of the amino acid residues in CDRs H1, H2, H3, L1 and L3 (Fig. 3a). An enlarged view of the complex exhibited well-defined electron density for the whole structure of the MUC1 glycopeptide 1 bearing ST antigen in the antigen binding site of SN-131 (Fig. 3b). The structure demonstrated occurrence of the direct interaction of SN-131 with the carbohydrate moiety of the MUC1 TRD-STs represented in the interactive surface structures, in which MUC1 glycopeptide 1 could be confirmed at the CDRs of the surface groove of the SN-131. The crystal structure analysis uncovered that the *O*-6 position of the GalNAc residue forms two hydrogen bonds with side chains of the amino acid residues in CDR H2 (His52 and Ser55 of the heavy chain) (Fig. 3c). As demonstrated by SPR experiments (Fig. 2d, e and Fig. S2, ESI†), these hydrogen bonds contribute strongly to the dramatically enhanced binding affinities with MUC1 TRD-Tn (3)/ST (4) by SN-131 in comparison to that with non-glycosylated MUC1 TRD (2). Interestingly, thermodynamic binding parameters determined by isothermal titration calorimetry (ITC) suggested that these enthalpically favourable hydrogen bonds with the *O*-6 position of the root GalNAc residue do not accompany any entropy penalty (Fig. S4, ESI†). These observations are consistent with the fact that sugar extensions at the *O*-6 position of the root GalNAc residue abrogates completely the binding ability of SN-131 with MUC1 TRD.^20^ Notably, the side chain of Asn35 in the CDR L1 forms a hydrogen bond with the *O*-9 position of the Neu5Ac residue of the ST antigen. This is the first 3D structure providing evidence that anti-MUC1 mAb interacts directly with the Neu5Ac moiety in the ST antigen attached to the proximal threonine residue within the immunodominant Asp-Thr-Arg motif. In addition, the binding between SN-131 and MUC1 glycopeptide 1 is stabilized by the interactions of the MUC1 core heptapeptide moiety, Ac-Pro1-Asp2-Thr3-Arg4-Pro5-Ala6-Pro7-NH_2_, with amino acid residues involved in the CDRs. As represented in Fig. 3d, the aspartic acid residue in the MUC1 glycopeptide 1 [Asp2 (MUC1)] forms hydrogen bonds with the main chain of Asn31 (H1), Gly101 (H3), and Thr102 (H3) in the SN-131 CDRs. It was uncovered that Arg4 (MUC1) contributes both to the hydrogen bonds with the side chains of Tyr59 (H2) and Ser99 (H3), and salt bridges with the side chain of Glu39 (L1) in CDRs, concurrently. In addition, Ala6 (MUC1) and Pro7 (MUC1) also form hydrogen bonds with the side chains of His31 (L1) and the main chain of Gly99 (L3), while CH/p interactions between the intra-cyclic methylene group of Pro5 (MUC1) and Pro7 (MUC1) and the two ring faces of His31 (L1) and Tyr59 (H2) may also contribute to the stabilization of the binding.

### Pancreatic cancer cells and secreted exosomes express MUC1 TRD having dynamic epitopes recognized by SN-131

**Fig. 4:**
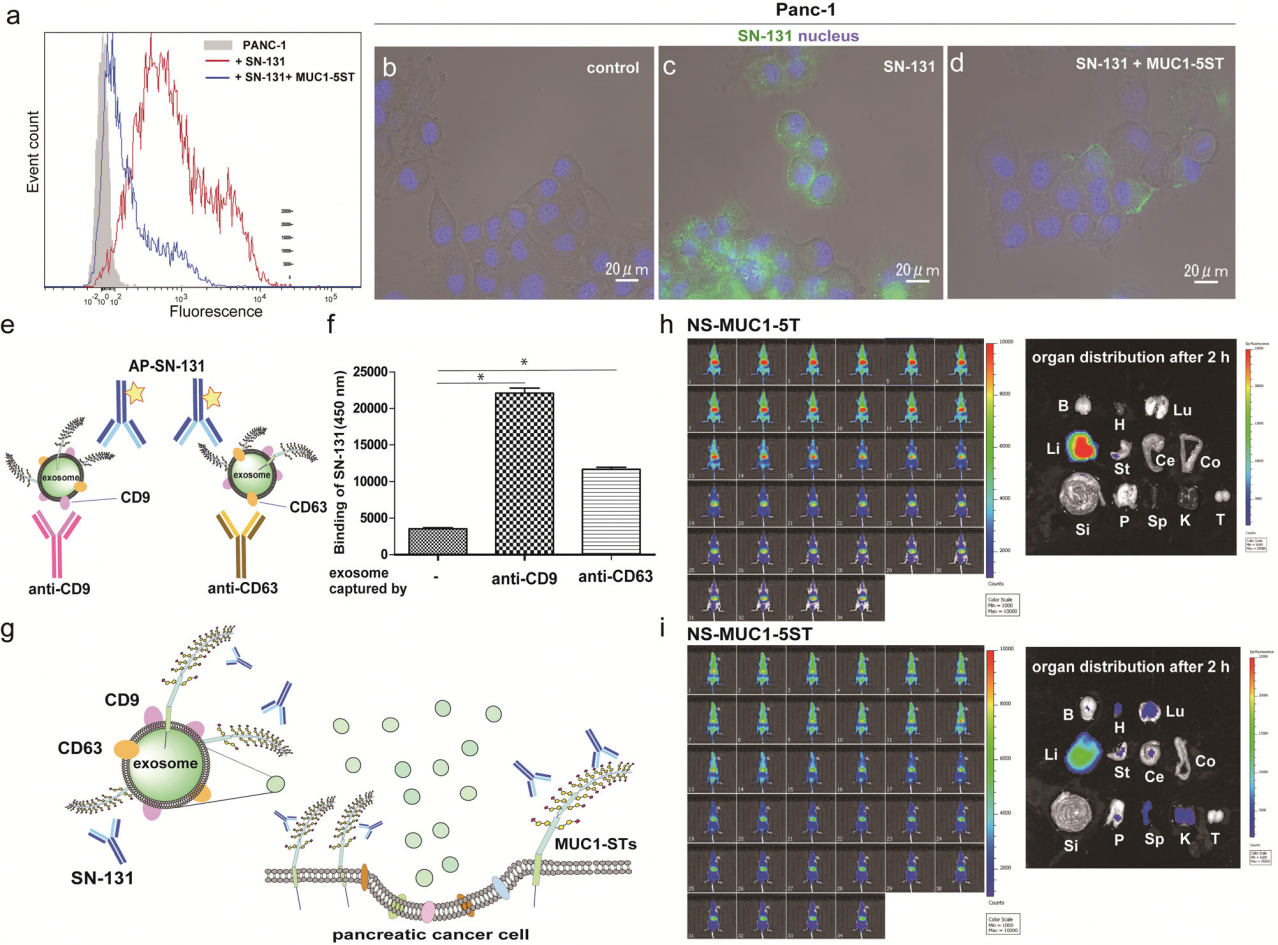
Pancreatic cancer cells and secreted exosomes express MUC1-TRDs recognized by SN-131. (a) Flow cytometry analysis of MUC1-ST expression levels in PANC-1 cells. Cells were incubated with Alexa488-conjugated SN-131 only (red line) or with 10 μM MUC1-5ST (8) (blue line). (b–d) Live cell image represents the significant binding of Alexa488-conjugated SN-131 with PANC-1 cells (c), while the binding is suppressed distinctly in the presence of 10 μM MUC1-5ST (8) (d). (e and f) Sandwich enzyme-linked immune-assay (ELISA) of the exosomes released from PANC-1 cells cultured in exosome-depleted FBS. Exosomes captured by antibodies to CD9 and CD63 were quantitated by alkaline phosphatase-labelled SN-131 in the presence of the substrate for alkaline phosphatase. *P < 0.001 (g) Human pancreatic cancer cells (PANC-1) release exosomes displaying MUC1 TRD containing dynamic epitopes of SN-131. Live animal NIR imaging of nanosomes (NSs) displaying MUC1-5T (7) (h) and MUC1-5ST (8) (i) at 0, 6, 12, 18, 24, 30, 36, 42, 48, 54 sec, and at 5, 10, 15, 20, 25, 30, 35, 40, 45, 50, 55, 60, 65, 70, 75, 80, 85, 90, 95, 100, 105, 110, 115, and 120 min after intravenous administration (left). Pictures representing organ biodistribution of NSs uncovered by dissection at 2 h after administration (right). Photo of isolated major organs such as brain (B), heart (H), lung (Lu), liver (Li), stomach (St), cecum (Ce), colon (Co), small intestine (Si), pancreas (P), spleen (Sp), kidney (K), and testis (T), respectively.

Flow cytometry analysis was conducted to determine whether pancreatic adenocarcinoma cancer cells express endogenous MUC1 TRD containing the “dynamic epitope” of SN-131. As shown in Fig. 4a, it was demonstrated that PANC-1 cells express a prominent level of MUC1 TRD modified with ST, T, and/or Tn antigens at the threonine residue involved in the immunodominant Asp-Thr-Arg region, while the binding is inhibited entirely by 10 μM MUC1-5ST (8). Similarly, live cell imaging analysis provided evidence that SN-131 binds strongly to PANC-1 cell surface MUC1 TRD (Fig. 4b and c), whereas SN-131 nearly lost the ability to engage PANC-1 cells in the presence of 10 μM MUC1-5ST (8) (Fig. 4d).

Strikingly, sandwich ELISA using anti-CD9/CD63 mAbs and SN-131 (alkaline phosphatase-labelled) that PANC-1 cells release displaying cargo MUC1 TRD recognized by SN-131 (Fig. 4e–g and Fig. S5, ESI†), implicating that exosomes secreted from pancreatic cancer cells could interact directly with immune cells expressing Siglecs. *In vivo* near infrared (NIR) imaging of nanosome (NS), an exosome model prepared from fluorescent light-emitting semiconductor quantum dots,^31,32^ displaying MUC1-5T (7) and MUC1-5ST (8) shed light on a pivotal role of the glycocalyx of MUC1 TRD without bias due to other components of exosomes (Fig. S6, ESI†). Our observations elicited that circulation, clearance, and organotropic biodistribution of exosomes derived from PANC-1 cells in mice depend strongly on the glycoforms of MUC1 TRD, particularly the difference of the terminal sugar residues between MUC1-5T (7) and 5ST (8) (Fig. 4h and i). Real-time NIR imaging and pictures of isolated organs by surgical dissection at 2 h revealed rapid and highly specific accumulation of NS-MUC1-5T in the liver after intravenous administration without distribution in any other organs evaluated (Fig. 4h). On the contrary, NS-MUC1-5ST was found to retain and distribute overall bodies during a 2 h longitudinal study, and pictures of isolated organs showed dramatically reduced liver accumulation and broad biodistribution of the NS-MUC1-5ST in the heart, lung, stomach, pancreas, spleen, and kidney (Fig. 4i). The results clearly indicate that MUC1 TRD with multiple ST antigens is crucial for distinctively prolonged circulation and broad organ biodistribution of exosomes secreted from pancreatic cancer cells. Although the underlying molecular mechanism determining differences in the body circulation and organotropism between NS-MUC1-5T and NS-MUC1-5ST remains unclear, we conceive that exosomes carrying MUC1 TRD-STs disseminated from the primary pancreatic cancer sites could contribute to organotropic cancer metastasis through the interaction with Siglecs of tissue-resident immune cells at the preferential pre-metastatic sites.

## Discussion

**Fig. 5:**
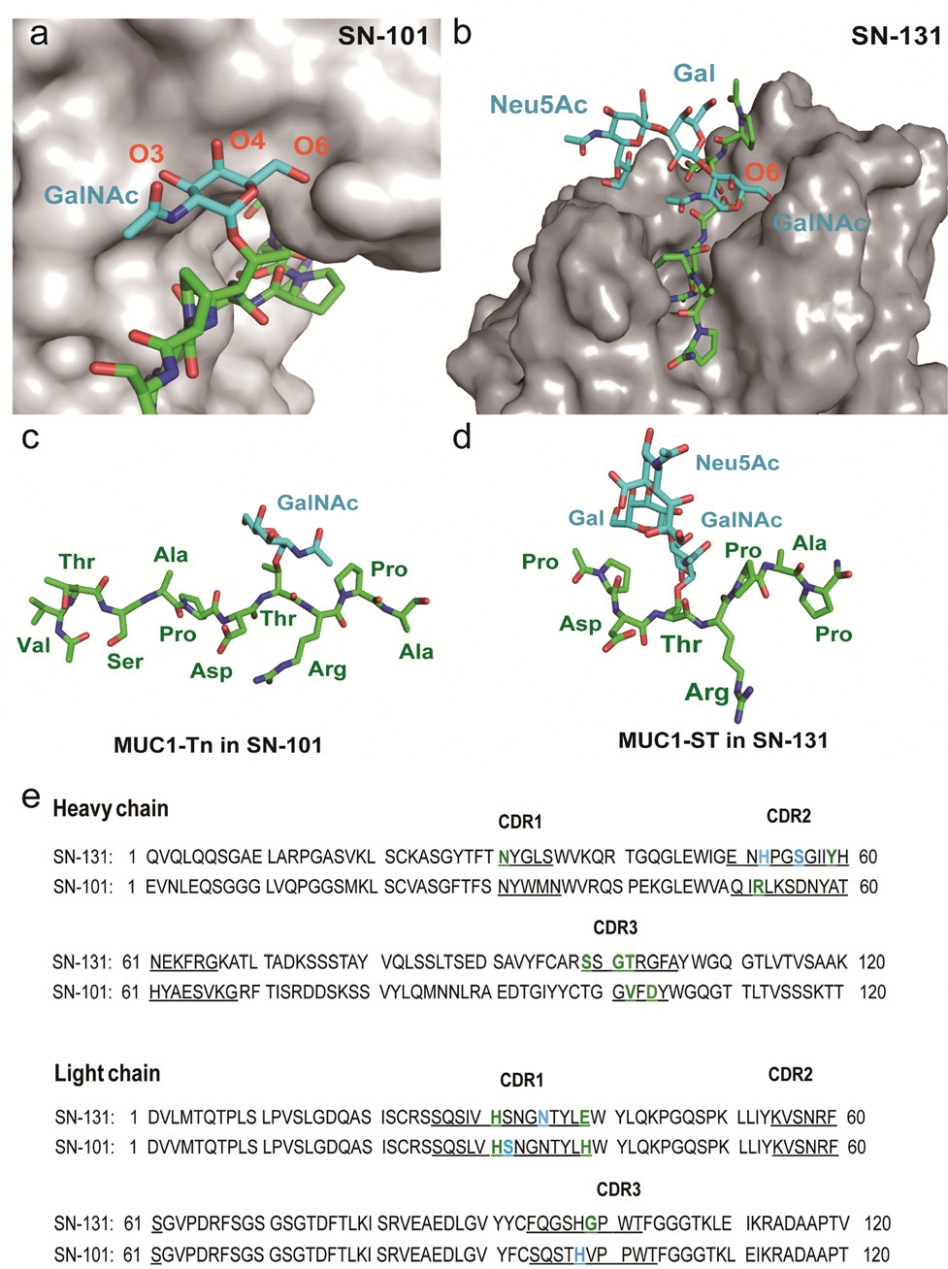
Distinctive molecular mechanisms in the recognition of dynamic epitopes by SN-101 and SN-131. (a) 3D crystal structure focusing on GalNAc recognition by SN-101 Fab in complex with MUC1-Tn, Ac-Val-Thr-Ser-Ala-Pro-Asp-Thr(Tn)-Arg-Pro-Ala-Pro-Gly-Ser-Thr-Ala-NH_2_ (PDB accession code: 6KX1).^20^ (b) 3D structure of the SN-131 surface represents the distinct topology of the binding cleft for MUC1-ST (1) obtained in the present study. MUC1-Tn bound to SN-101 (c) and MUC1-ST (1) bound to SN-131 (d), focusing on the difference in the conformations of the proximal peptide moieties observed in the combined structures, respectively. (e) Amino acid sequences of variable regions of anti-MUC1 mAbs SN-131 and SN-101. The underlined letters are amino acids in CDRs. Residues that interact with carbohydrate moieties are colored with cyan and residues that interact with peptide moieties are colored with green, respectively.

The present results demonstrate the importance of understanding the structural and molecular basis in the recognition of glycopeptidic “dynamic epitopes” in the MUC1 TRD by the anti-MUC1 mAbs. Notably, SN-131 interacts specifically with an immunodominant Pro-Asp-Thr-Arg moiety having Tn/T/ST antigens independent of the occupation status at 4 other potential *O*-glycosylation sites. Clearly, MUC1 TRD-STs highly expressed on the PANC-1 cell surface can be counter receptors (epitopes) simultaneously both of Siglecs and SN-131, implicating that SN-131 may abrogate interaction of Siglecs with PANC-1 cells. Importantly, SN-131 binds tightly both MUC1-ST (4) and MUC1-5ST (8), while there is no space for the interaction of SN-101 with longer and complex *O*-glycans modified at both C-3 and C-6 positions of the GalNAc residue (Fig. 5a).^20^ In contrast, the X-ray structure of SN-131 complexed with MUC1-ST (1) represents the interactive surface structure adapting to core 1 type *O*-glycans such as T and ST antigens in addition to the GalNAc residue (Fig. 5b).

Interestingly, two dynamic epitopes bound to the antibodies SN-101 and SN-131 were found to form distinctive conformations, suggesting that the difference in enthalpy/entropy compensation during the complex formation is pivotal for the determination of the binding specificity and strength in antibody recognition (Fig. 5c, d and Fig. S4, ESI†).^33^ It seems likely that hydrogen bonds between MUC1-ST (1) and amino acids in CRDs of SN-131 produce a “turn-like” structure through an induced fitting mechanism (Fig. 5d and e), while Arg52H, Val102H, His31L, His39L, and Asp104L in CDRs of SN-101 have been found to contribute to the formation of a “rigid extended” structure (Fig. 5c and e).^22^ The GalNAc addition seems to give advantages to the binding by overcoming the entropy-enthalpy compensation problem (Fig. S4, ESI†). However, we could not find significant hydrophobic interactions between GalNAc and SN-131, suggesting that there is some new factor for decreasing the −*T*Δ*S* value. Our observations elicit that occupation states and glycoforms in the five potential *O*-glycosylation sites in MUC1 TRD^24,34^ strongly influence the inhibitory effect by anti-MUC1 mAbs on the anti-cancer activity through interaction with key partner molecules.

We also found that exosomes secreted from cultured PANC-1 cells also carry MUC1 TRD-STs by sandwich ELISA using SN-131 and anti-CD9/CD63 mAbs. This finding implies that adhesion of Siglecs with exosomes released from the parent PANC-1 cells might affect the formation of the pre-metastatic niche. Notably, tumour-derived exosomes are emerging as critical mediators of intercellular communication between cancer cells and stromal cells both in local and distant cancer microenvironments.^35,36^ There is also evidence showing that exosomes derived from pancreatic cancer cells play crucial roles in the formation of the liver pre-metastatic niche.^37^ It is conceivable that pancreatic cancer cell-derived exosomes displaying MUC1 TRD-STs could transfer horizontally their cargo to immune cells and stromal cells by engaging with various Siglecs such as Siglec-4a, Siglec-7, Siglec-9, and Siglec-10.^7,11,29,30^

## Conclusions

We demonstrated that SN-131 is a promising antibody that exhibits specific and strong binding affinity with pancreatic cancer-relevant MUC1 TRDs carrying Tn and core 1 type *O*-glycans such as T and particularly ST antigens at the immunodominant Asp-Thr-Arg motif. The merit of this antibody is evident because normal cells express MUC1 modified dominantly with mature core 2 type *O*-glycans.^15,28^ Our results elicited the importance of understanding the structural and molecular basis in the antibody recognition of site-specifically glycosylated neoepitopes, which is indispensable for preventing the off-targeting effect by the antibody-based anticancer therapy. Undoubtedly, the strategy for the development of novel antibodies recognizing cancer-specific “dynamic neoepitopes” elaborated by site-specific glycosylation will open up new avenues for early detection and therapeutic intervention for pancreatic cancer progression, recurrence, and metastasis. Further biological and anticancer activities of SN-131 and antibody drug conjugates derived from this antibody are under way and the results will be reported as soon as possible.

## Experimental

### Synthesis of MUC1 TRD glycopeptides

Chemical and enzymatic synthesis of MUC1 TRD glycopeptides 1–9 was basically performed according to the standard method established at the Hokkaido University^21–24,38–40^ with suited modifications in the *N*- and *C*-terminal functionalization required. Microwave-assisted solid-phase synthesis was carried out with an EYELA microwave synthesizer Wave Magic MWS-1000A (Tokyo Rikakikai, Tokyo) at 50 °C on Rink amide ChemMatrix resin (loading 0.54 mmole g^−1^) by means of *N*α-Fmoc-amino acid derivatives and glycosylated *N*α-Fmoc-amino acid derivatives, such as *N*α-Fmoc-Thr(GalNAcα1 →)-OH, *N*α-Fmoc-Thr(Galβ1,3GalNAcα1 →)-OH and *N*α-Fmoc-Thr(Galβ1,3GalNAcα1 →)-OH. Detail synthetic procedures and characterisation data are described in the ESI.†

### Glycopeptide microarray

100 mM solutions of the synthetic glycopeptides capped with ketone-linker at the *N*-terminus (5–9) dissolved in 25 mM sodium acetate buffer (pH 5.0) containing 0.005% Tween-20 (w/v) were robotically printed on plastic slides coated with a copolymer carrying aminooxy-/phosphorylcholine functional groups according to the protocol reported previously.^20,22,24^ Detailed conditions for the binding assay are described in the ESI.†

### Anti-MUC1 mAb (SN-131)

Hybridoma producing anti-MUC1 mAb (SN-131/1B2)^19^ was cultured with SFM4MAb w/L-Gln (1 L, cytiva) containing 2% FBS in a culture bag (A-1000NL, NIPRO) at 37 °C under 5% CO_2_ atmosphere. Procedures and conditions for the preparation and purification of SN-131 Fab were described in the ESI.†

### Binding affinity of SN-131 Fab with MUC1 TRD models

The binding affinity of SN-131 Fab with MUC1 TRD models was measured by using a Biacore 2000 surface plasmon resonance (SPR) instrument (GE Healthcare). Synthetic MUC1 TRD models 2–4 with a *C*-terminal cysteine residue were immobilized on a CM5 chip (GE Healthcare) by a standard protocol of the maleimide coupling. The SN-131 Fab was injected over the surface of the immobilized MUC1 TRD models 2–4 and three kinetic parameters, the association rate constant (kon), the dissociation rate constant (koff), and the equilibrium dissociation constant (*K*_D_) were obtained with BIAevaluation 4.1 software (GE Healthcare) using a bivalent binding model. Isothermal titration calorimetry (ITC) experiments were also carried out to determine the thermodynamic parameters of the interaction between SN-131 and non-glycosylated-/Tn-MUC1 TRDs.7 All experiments and analyses were performed by using nano ITC (TA Instrument) and Nanoanalyze based on as independent model.

### Crystallization of SN-131 Fab/MUC1-ST

SN-131 Fab was subjected to treatment with 1 M dimethylamine-borane complex and 1 M formaldehyde for the methylation of Lys residues according to the method reported previously.^41^ After purification by size exclusion chromatography using HiLoad 16/600 Superdex 75 column (GE Healthcare) in 10 mM Tris-HCl buffer containing 100 mM NaCl (pH 8.0), the solution was concentrated to 30 mg mL^−1^ and mixed with MUC1-ST (1) at a 1 : 4 molar ratio. Crystallization of SN-131 Fab was performed using the sitting-drop vapor-diffusion method with JCSG core suites (Qiagen, Germany). Antibody solution (0.2 μL) was mixed with an equal volume of reservoir solution and incubated at 20 °C for 8 months. High quality crystals were obtained from a solution composed of 40% (w/v) PEG 300, 5% (w/v) PEG 1000, and 0.1 M Tris-HCl buffer (pH 7.0).

### Data collection, structural determination, and refinement

Crystals were soaked in cryo-protectant solution (20% glycerol in reservoir solution), followed by flash-cooling under a stream of liquid nitrogen at −183 °C. Diffraction data were collected on beamline BL1A at the Photon Factory (Tsukuba, Japan). All data were processed and scaled using XDS.^42^ All data collection statistics are summarized in (Table S1, ESI†). The structures of SN-131 Fab were determined by the molecular replacement method with the program PHASER.^43^ Several rounds of refinement were performed using the program Phenix refin^44^ in the Phenix program suite, alternating with manual fitting and rebuilding based on 2Fo–Fc and Fo–Fc electron density using COOT.^45^ Then, water molecules and MUC1-ST (1) were built based on 2Fo–Fc and Fo–Fc electron densities. The final refinement statistics and geometry defined by using MolProbity^46^ are shown in (Table S1, ESI†). All structural figures were generated by using PyMol (W. L. DeLano, The PyMOL Molecular Graphics System, Version 1.7.4 Schrödinger, LLC, 2002).

### Cancer cell culture and flow cytometry analysis

The human pancreatic cancer cell line PANC-1 (CRL-1469) acquired from ATCC was cultured in Dulbecco's modified Eagle's medium (D-MEM, low glucose) supplemented with 10% FBS and 2 mM l-glutamine in 5% CO_2_ at 37 °C. For the flow cytometry, the PANC-1 cells cultured for 48 h were trypsinized using 0.25% trypsin/EDTA solution and washed with the D-MEM medium and PBS/EDTA (D-PBS containing 2 mM EDTA). PANC-1 cells (1 × 10^6^ cells) were suspended in 10 mg mL^−1^ of Alexa 488 conjugated SN-131 of 10 mM MUC1-5ST (8) in the PBS/EDTA solution. After 2 h incubation on ice, cells were washed with PBS/EDTA twice. The analysis was performed by using FACS Canto (BD Bioscience) and the obtained data were analysed by FCSalyzer Version 0.9.22-alpha (Sven Mostb).

### Live cell imaging

PANC-1 cells (5 × 10^4^ cells per well) were cultured with 10% FBS and 2 mM l-glutamine on an 8-well imaging plate (Fischer Scientific) for 48 h in 5% CO_2_ at 37 °C. Then, Alexa 488 conjugated SN-131 (10 μg mL^−1^), 10 μM MUC1-5ST (8), or MAG (5 μg mL^−1^) dissolved in Opti-MEM medium (gibco) was co-incubated with PANC-1 cells for 2 h in 5% CO_2_ at 37 °C. Cells were washed with Opti-MEM and stained with Hoechst 33342 (2.5 ng mL^−1^ in Opti-MEM) for 30 min. After washing with Opti-MEM twice, the stained cells were observed with an all-in-one fluorescence microscope (BIOREV BZ-9000 series generation II, Keyence, Osaka, Japan). The pictures were imaged with 60xlens and the following filter set (Blue: #OP-66843 Excitation 360/40, Emission 460/50, Green: #OP-66836, Excitation 470/40, Emission 535/50, Red: #OP-66837, Excitation 540/25, Emission 605/550). Imaging pictures were analysed by BZ-II Analyzer software (Keyence, Osaka).

### Exosome isolation from pancreatic cancer cells

PANC-1 cells were cultured in D-MEM supplemented with 10% exosome-depleted FBS (FBS, System Biosciences) until the cells reached 70–80% confluency in a 150 mm dish and maintained in a humid incubator with 5% CO_2_ for 2 days at 37 °C. Supernatant fractions collected from 6 dishes were centrifuged at 300 g for 10 min at 4 °C. The supernatant was centrifuged twice (at 2000 g for 10 min and at 10 000 g for 30 min at 4 °C) to remove the precipitate. The supernatant containing crude exosomes were then collected by ultracentrifugation at 100 000 g for 70 min at 4 °C. The exosome pellet was resuspended in D-PBS and collected by ultracentrifugation at 100 000 g for 70 min at 4 °C (Beckman Coulter L-80XP, SW-32Ti). Exosome size and numbers were analysed using the LM10 nanoparticle characterization system (Nano Sight, Malvern) equipped with a blue laser (405 nm).^36^

### Sandwich enzyme-linked immunosorbent assay of exosomal MUC1 TRD-STs

Anti-CD9 mAb and anti-CD63 mAb (Fujifilm Wako Chemicals) in PBS (1/500 dilution, 100 μL) were coated on 96-well enzyme-linked immunosorbent assay plates. The plates were sealed and incubated overnight at 4 °C, then washed twice with 25 mM Tris-HCl buffer containing 150 mM NaCl, and 0.1% BSA. After blocking with 1% BSA in PBS (200 μL) for 4 h at 4 °C, exosome solution isolated from PANC-1 cells (1/1000 dilution, ca 0.5 pM) was added to the plates and incubated for 2 h at room temperature. Plate was washed and AP (alkaline phosphatase) labelled SN-131 mAb (1 μg mL^−1^, 100 μL) was added and incubated for 2 h at room temperature. After washing with PBS, CDP-Star AP Substrate (Merck, #69086) was used for colour development. After incubating for 30 min at 37 °C, luminescence was determined at 450 nm with a plate reader (SpectraMaxM5, Molecular Device) and the data were analysed using Graphpad Prism 6 software.

### Live animal NIR imaging of exosome models

Nanosomes (NSs) displaying MUC1-5T (7) and MUC-5ST (8) were prepared from *tri*-octyl phosphine oxide (TOPO)-coated quantum dots (800 nm, Thermo Fischer Scientific) using 11,11′-dithio bis[undec-11-yl 12-(aminooxyacetyl)aminohexa(ethyleneglycol)] (AO-SH) and 11-mercaptoundecylphosphorylcholine (PC-SH) according to a method reported previously.^17,18^ In brief, 1 μM NSs (250 μL, AO/PC = 1/25) were added to the solution of 1 mM MUC1-5T (7) or MUC1-5ST (8) (50 μL) in 100 mM acetate buffer (100 μL, pH 5.0) and the mixture was concentrated to dryness using a centrifugal evaporator to complete the oxime formation for 16 h. The dried NSs were dissolved with MilliQ, purified by ultrafiltration with Amicon ultra 0.5 (Millipore), and dissolved in 0.9% NaCl in water (250 μL). NSs was characterized by nanoparticle tracking analysis using Nano Sight (Malvern).

NS-MUC1-5T (ca 50 nm) and NS-MUC1-5ST (ca 60 nm) (100 μL, 1 μM/saline) were injected into the tail veins of BALB/c slc-nu/nu (7-week-old, male, *n* = 2; Sankyo Lab. Service, Tokyo, Japan) suited for sensitive fluorescence measurements in the NIR imaging without the influence of the body hair. Then, the mice were anesthetized by isoflurane, and were imaged using an IVIS imaging system (Summit Pharmaceuticals International Co., Tokyo, Japan). The exposure time is 1 sec., and the wavelength of excitation and emission is 710 nm and 820 nm, respectively. The mice were imaged for 2 h after intravenous injection. After 2 h imaging, the mice were dissected, and the fluorescence images were taken. Major organs were isolated from mice and organ fluorescence images were also observed according to the method described in a previous report.^17,18^ Live animal imaging experiments using mice were approved by the Institutional Animal Care and Use Committee of Hokkaido University and performed in accordance with ARRIVE guidelines and regulations of this committee (National University Corporation Hokkaido University Regulations on Animal Experimentation).

### Statistical analysis

Error bars in the graphical data represent means ± s.e.m. Statistical significance was determined using a two-tailed Student's *t*-test or by ANOVA. *P* < 0.001 was considered statistically significant. Statistical analyses were performed using GraphPad Prism software.

## Data availability

The X-ray crystallographic coordinates and structure factor file of the anti-MUC1 mAb (SN-131)/MUC1 glycopeptide (1) have been deposited in the Protein Data Bank (PDB) with the accession code 8HRH. The data that support the findings of this study are available from the corresponding author upon request.

## Conflicts of interest

There are no conflicts to declare.

## Supplementary Material

CB-004-D3CB00036B-s001
