# Supplementary material for: Structural and molecular insight into antibody recognition of dynamic neoepitopes in membrane tethered MUC1 of pancreatic cancer cells and secreted exosomes
Source: RSC Chem Biol. 2023 May 24;4(8):564–72. doi: 10.1039/d3cb00036b (PMC10398351; doi:10.1039/d3cb00036b)
Supplement: CB-004-D3CB00036B-s001 [file CB-004-D3CB00036B-s001.pdf]

Electronic Supplementary Material (ESI) for RSC Chemical Biology.
This journal is © The Royal Society of Chemistry 2023

[Supplementary Information]

Structural and molecular insight into antibody recognition of dynamic neoepitope in membrane tethered MUC1 of pancreatic cancer cells and secreted exosomes

Hajime Wakui,[a] Yasuhiro Yokoi,[a] Chieko Horidome,[a] Toyoyuki Ose,[b] Min Yao,[b] Yoshikazu Tanaka,[c] Hiroshi Hinou,[a] and Shin-Ichiro Nishimura*[a]

[a]*Field of Drug Discovery Research, Faculty of Advanced Life Science, and Graduate School of Life Science, Hokkaido University, N21, W11, Kita-ku, Sapporo 001-0021*

[b]*Field of X-ray Structural Biology, Faculty of Advanced Life Science, and Graduate School of Life Science, Hokkaido University, N10, W8, Kita-ku, Sapporo 060-0810*

[c]*Graduate School of Life Science, Tohoku University, 2-1-1 Katahira, Aoba-ku, Sendai 980-8577, Japan*

Corresponding author: Shin-Ichiro Nishimura, e-mail: shin@sci.hokudai.ac.jp

METHODS

Reagents and general methods. All commercially available solvents and reagents were used without further purification. Rink Amide-ChemMatrix resin was purchased from Biotage Japan Ltd. (Tokyo, Japan). *N*α-Fmoc-amino acid derivatives except for glycosylated amino acid derivatives were purchased from Watanabe Chemical Industries, Ltd. (Hiroshima, Japan). *N*α-Fmoc-Thr(Ac_2GalNAcα1→)-OH, *N*α-Fmoc-Thr(Ac_4Galβ1,3Ac_2GalNAcα1→)-OH, and *N*α-Fmoc-Thr(Ac_4Galβ1,3Ac_2GalNAcα1→)-OH were synthesized basically according to the procedures reported previously with some modifications.[1] 1-[Bis(dimethylamino)methyliumyl]-1H-benzotriazole-3-oxide hexafluorophosphate (HBTU), 1-hydroxybenzotriazole monohydrate (HOBt), *N,N*-diisopropylethylamine (DIEA), *N,N*-dimethylformamide (DMF) and 2,2,2-trifluoroacetic acid (TFA) were purchased from Watanabe Chemical Industries, Ltd. (Hiroshima, Japan). All mixing operation in peptide synthesis was performed by a vortex mixer. All solid-phase reactions for (glyco)peptides synthesis were performed manually in a polypropylene tube equipped with a filter (LibraTube, Hipep Laboratories) under microwave irradiation. The microwave was irradiated during coupling reactions and Fmoc removal using temperature control at 50˚C. Matrix-assisted laser desorption/ionization time-of-flight mass spectrometry (MALDI-TOFMS) were recorded with a Bruker UltraFlex III mass spectrometer in reflector positive or linear negative mode using matrix as 2,5-dihydroxybenzoic acid (DHB). Typically, the samples were dissolved in 1 µL of 50 % (v/v) aqueous acetonitrile and mixed with the same volume of 10 mg/mL DHB in 50 % (v/v) aqueous acetonitrile containing 0.1 % TFA. A reverse-phase HPLC (RP-HPLC) analysis was performed on a Hitachi system equipped with L-6250 intelligent pump and L-7400 UV detector using a reverse-phase C18 column Inertsil ODS-3 250×20 mmI.D. (GL Sciences Inc., Tokyo, Japan) or Shimadzu HPLC system (Shimadzu Corporation, Kyoto, Japan) with LC-20B pump, SPD-20A UV/VIS detector at 220 nm for monitor using Inertsil ODS-3 reversed-phase C-18 column C18 ODS-3 250×20 mm I.D (GL Sciences Inc., Tokyo, Japan). A preparative RP-HPLC was performed on a Hitachi system equipped with L-7100 pump and L-7405 UV detector using reverse-phase C18 column Inertsil ODS-3 250×4.6 mmI.D. (GL Sciences Inc., Tokyo, Japan). Protein purification was performed by AKTA explorer

100 (GE healthcare) at a 4°C environment. Protein concentration was measured by Nanodrop 2000 (Thermo Scientific). Crystallization screening was carried out by the sitting-drop vapor-diffusion method against JCSG core suits, using mosquito (AS ONE, Japam). Drops consisting of 0.2 μL of protein and an equal volume of reservoir buffer were equilibrated against 75 μL of reservoir solution. CM5 sensor chip, HPS-EP buffer and Glycine HCl buffer (pH2.0) were purchased from Cytiva. Microarray slides (75×25×1 mm) were supplied from Sumitomo Bakelite Co., Ltd. (Tokyo, Japan). Spotting of glycopeptide derivatives was performed using ChipMaker™ with CMP6 micro spotting pins. Cy3-labeled goat anti-mouse IgG antibody was purchased from Jackson ImmunoResearch (Pennsylvania, USA). Fluorescence of microarray slides was detected using GlycoStation reader 1200 (GlycoTechnica Ltd., Yokohama, Japan) and processed using ArrayVision software version 8.0 (GE Healthcare). Statistical analysis and graphing were performed by Graphpad Prism software GraphPad Prism 7 software (GraphPad, La Jolla, CA)

Synthesis of MUC1 TRD glycopeptides. Chemical and enzymatic synthesis of MUC1 TRD and FN model glycopeptides **1~9** was basically performed according to the standard method established at the Hokkaido University[2-6] with suited modifications in the *N*- and *C*-terminal functionalization required. Microwave-assisted solid-phase synthesis was carried out with an EYELA microwave synthesizer Wave Magic MWS-1000A (Tokyo Rikakikai, Tokyo) at 50°C on Rink amide ChemMatrix resin (loading 0.54 mmole/g) by means of *N*α-Fmoc-amino acid derivatives and glycosylated *N*α-Fmoc-amino acid derivatives, such as *N*α-Fmoc-Thr(GalNAcα1→)-OH, *N*α-Fmoc-Thr(Galβ1,3GalNAcα1→)-OH and *N*α-Fmoc-Thr(Galβ1,3GalNAcα1→)-OH. Prior to use, the resin was swollen with dichloromethane at ambient temperature for 2 h and washed with DMF. For the coupling of common *N*α-Fmoc-amino acids, *N*α-Fmoc-amino acid derivatives (4.0 eq.) was coupled with HBTU (4.0 eq.), HOBt (4.0 eq.), and DIEA (6.0 eq.) in DMF for 9 min at 50°C under microwave irradiation. In case for glycosylated *N*α-Fmoc-amino acid derivatives, *N*α-Fmoc-Thr(GalNAcα1→)-OH or *N*α-Fmoc-Thr(Galβ1,3GalNAcα1→)-OH (1.5 eq.), HBTU (1.5 eq.), HOBt (1.5 eq.), and DIEA (3.0 eq.) in DMF for 9 min at 50°C under microwave irradiation. Removal of the Fmoc group was performed

with 20 % piperidine in DMF at 50°C for 3 min. Washing steps between Fmoc group-deprotection, coupling and subsequent deprotection steps were performed at room temperature with DMF (3 x 1min), dichloromethane (3 x 1min) and DMF (3 x 1 min). Unreacted amino groups generated at each coupling step including the termination of compounds **1~4** were capped by acetylation with Ac_2O/DIEA/DMF solution (10 : 5 : 85, v/v/v, 1 mL) for 5 min at room temperature. Compounds **5~9** were terminated by coupling with 5-oxohexanoic acid (3.0 eq.). Cleavage of glycopeptides and intermediates from resin and removal of side chain protective groups were performed by treatment with TFA/H_2O/triisopropylsilane (95 : 2.5 : 2.5) at room temperature for 2 h, while compounds containing both 5-oxohexanoyl group and *C*-terminal cysteine residue such as **2**, **3**, **4**, **7**, **8** and **9** were treated with TFA/thioanisole/phenol/1-dodecanethiol (82.5 : 5 : 5 : 2.5). Finally, the resin was filtered off, and the glycopeptides were precipitated using *tert*-butyl methyl ether in an ice bath. After centrifugation (3000 rpm at 4°C for 10 min), the supernatant was carefully removed. For the precipitate of non-glycosylated peptides **2** and **5** was dissolved in 50% acetonitrile in water and lyophilized. In case of other glycopeptides, the precipitate was dissolved in sodium methoxide/methanol (pH12.5) and kept for 30 min at an ambient temperature to remove *O*-acetyl groups of the sugar moiety. The solution was neutralized with 30% acetic acid in methanol and the solvent was removed by evaporation. The residual materials corresponding to glycopeptides having asialo-*O*-glycans such as **3**, **6**, and **7** were purified by using reverse-phase high-performance liquid chromatography (RP-HPLC).

Precursors of the compounds **1**, **4**, **8**, and **9** were subjected directly to the enzymatic sialylation with recombinant rat α2,3-(*O*)-sialyltransferase (CalBioChem) or recombinant *Pasteurella multocida* α2,3-sialyltransferase (Sigma Aldrich) in the presence of excess CMP-Neu5Ac. A typical condition used for the synthesis of compound **8** is as follows: 2 mM MUC1 TRD-5T (**7**) (3.1 mg, 0.8 mmole) was incubated with recombinant rat α2,3-(*O*)-sialyltransferase (150 mU/mL) in the presence of 30 mM CMP-Neu5Ac in a total volume of 400 mL of HEPES buffer containing 10 mM of $MgCl_2$ and 0.1 % BSA (pH7.0) for 24 h at 2°C. The reaction mixture was subjected to the purification by preparative RP-HPLC and the pure fraction, as confirmed by mass spectrometry, was lyophilized to yield MUC1 TRD-5ST (**8**) in 63% (2.7 mg, 0.5 mmole). HPLC profile and MALDI-TOFMS of all new compounds such as **1**, **2**, **4**, **7**, **8**, and **9** were shown

as Additional Data in Supporting Information. Compounds **3**, **5**, and **6** were synthesized previously[5,7,8] and available for the present study.

Glycopeptide microarray. 100 mM solutions of the synthetic glycopeptides capped with ketone-linker at *N*-terminus (**5~9**) dissolved in 25 mM sodium acetate buffer (pH 5.0) containing 0.005% Tween-20 (w/v) were robotically printed on plastic slides coated with a copolymer carrying aminooxy-/phosphorylcholine functional groups according to the protocol reported previously.[5,7-9] For the epitope analysis of SN-131, antibody solutions (1 μg/mL) were added to each well of the slides. After incubation for 2 h at room temperature, the slides were rinsed and added with Cy3 labeled anti-mouse IgG polyclonal antibody (1 μg/mL). After washing, fluorescence of the wells were measured using a GlycoStation 1200 system and analysed with the ArrayVision software.

Anti-MUC1 mAb (SN-131). Hybridoma producing anti-MUC1 mAb (SN-131/1B2)[10] was cultured with SFM4MAb w/L-Gln (1 L, cytiva) containing 2% FBS in a culture bag (A-1000NL, NIPRO) at 37˚C under 5% CO_2 atmosphere. After 24 h, the supernatants were collected, centrifuged at 5000G, filtrated with 0.45 μm filter, and subjected to the affinity chromatographic purification on the basis of the MUC1 glycopeptide immobilized on a HiTrap NHS-activated HP column (cytiva) to yield a pure SN-131 fraction (23 mg/L).

To obtain SN-131 Fab, 38.7 mL of SN-131 IgG (1 mg/mL) was dialyzed against 1.5 L of 50 mM EDTA, 25 mM cysteine, and 10 mM citrate solution (pH 6.5). The mixture was concentrated to 10 mL and added to 1.5 mL of immobilized Ficin resin (Thermo Scientific) and shaken for 5 h at 37˚C. The reaction mixture was filtered to remove the resin and dialyzed into 40 mM sodium acetate buffer (pH 5.0). Fab fragments were purified by cation exchange chromatography on 1 mL of RESOURCE column (GE Healthcare) to afford pure SN-131 Fab fractions. The fractions containing Fab fractions were collected and concentrated to 3 mL using filter units of 30,000 MWCO (Millipore) and followed by size-exclusion chromatography on HiLoad 16/600 Superdex 75pg column (GE Healthcare) in 10 mM Tris-HCl buffer containing 100 mM NaCl (pH 8.0).

References for Supplementary Information

1. Y. Tachibana, *et al*. Antifreeze glycoproteins: Elucidation of the structural motifs that are essential for antifreeze activity. *Angew. Chem. Int. Ed*. **2004**, 43, 856-862.

2. T. Matsushita, *et al*., Rapid microware-assisted solid-phase glycopeptide synthesis. *Org. Lett*. **2005**, 7, 877-880.

3. M. Fumoto, *et al*., Combinatorial synthesis of MUC1 glycopeptides: Polymer blotting facilitates chemical and enzymatic synthesis of highly complicated mucin glycopeptides. *J. Am. Chem. Soc*. **2005**, 127, 11804-11818.

4. N. Ohyabu, *et al*., An essential epitope of anti-MUC1 monoclonal antibody KL-6 revealed by focused glycopeptide library. *J. Am. Chem. Soc*. **2009**, 131, 17102-17109.

5. T. Matsushita, *et al*., A straightforward protocol for the preparation of high-performance microarray displaying synthetic MUC1 glycopeptides. *Biochim. Biophys. Acta Gen. Subj*. **2014**, 1840, 1105-1116.

6. N. Ohyabu, *et al*., Convergent solid-phase synthesis of macromolecular MUC1 models truly mimicking serum glycoprotein biomarkers of interstitial lung diseases. *J. Am. Chem. Soc*. **2016**, 138, 8392-8395.

7. H. Wakui, *et al*., A straightforward approach to antibodies recognizing cancer specific glycopeptidic neoepitopes. *Chem. Sci*. **2020**, 11, 4999-5006.

8. S. Rangappa, *et al*., Effects of the multiple *O*-glycosylation states on antibody recognition of the immunodominant motif in MUC1 extracellular tandem repeats. *Med. Chem. Commun*. **2016**, 7, 1102-1122.

9. G. Artigas, *et al*., Glycopeptides as targets for dendritic cells: Exploring MUC1 glycopeptides binding profile toward macrophage galactose-type lectin (MGL) orthologs. *J. Med. Chem*. **2017**, 60, 9012-9021.

10. S. Naito, *et al*., Generation of novel anti-MUC1 monoclonal antibodies with designed carbohydrate specificities using MUC1 glycopeptide library. *ACS Omega* **2017**, 2, 7493-7505.

Table S1 | Structural data collection and refinement of SN-131 bound to MUC1-ST (**1**)

Data collection	SN-131/MUC1-ST (**1**) (PDB ID: 8HRH)
Space group	P6522
Cell dimensions	
a, b, c (Å)	84.59, 84.59, 271.27
a, b, g (°)	90.0, 90.0, 120.0
Resolution (Å)	2.07~49.766 (2.07~2.20)
R_{meas} (%)	12.7 (75.8)
I/sigma	16.60 (3.43)
CC1/2	99.9 (95.6)
Completeness (%)	99.9 (99.7)
Redundancy	16.6
No. of unique reflections	36063
R_{work} / R_{free} (%)	0.1919/0.2292
No. atoms	
Protein	3376
Ligands	110
Water	220
B-factors (Å^2)	
Protein	37.25
Ligands	48.35
R.m.s. deviations	
Bond lengths (Å)	0.005
Bond angles (°)	0.862
Ramachandran plot statistics	
Most favorable (%)	97.04
Outliers (%)	0.54
Clash score	5.37

Values in parentheses are for highest-resolution shell.

$$R_{meas} = \frac{\sum_{hkl}\sum_{i=1}^{n}|I_i(hkl) - \bar{I}(hkl)|}{\sum_{hkl}\sum_{i=1}^{n} I_i(hkl)}$$

I_i (hkl) is the mean intensities of a set of equivalent reflections.

$$R_{work}(\%) = \frac{\sum_{hkl}\big||F_{obs}(hkl)| - |F_{calc}(hkl)|\big|}{\sum_{hkl}|F_{obs}(hkl)|}$$

R_{free}(%) = R_{work} calculated using 5% of the reflection data chosen randomly and omitted from the refinement.

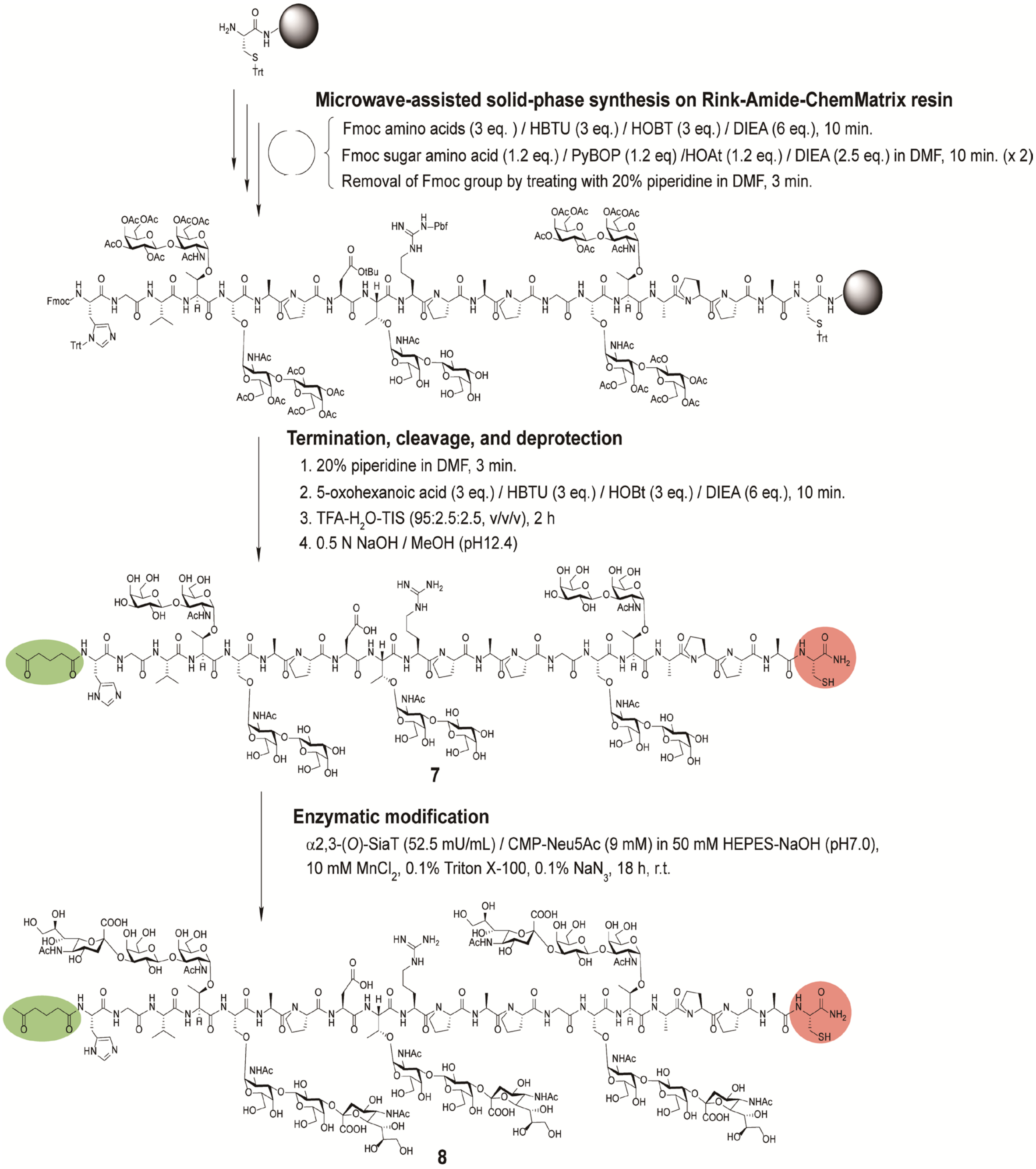

Scheme S1 | Synthetic protocol for compounds 7 and 8. A typical example of the synthesis of bi-functional MUC1 TRD glycopeptides **7** and **8** terminated by 5-oxo group at *N*-terminus and *C*-terminal cysteine residue, concurrently. These glycopeptide derivatives can be immobilized covalently with surfaces of the microarray, SPR chip, ELISA plates and nanosome as an artificial exosome in addition to the conjugation with protein carriers.

a

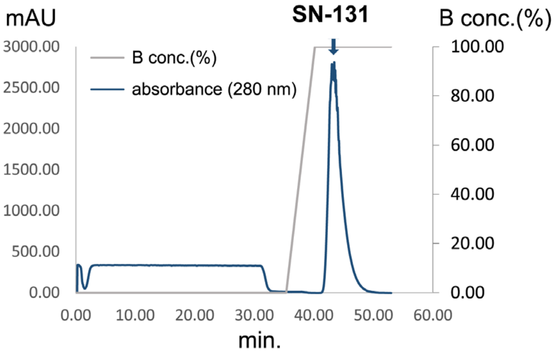

b

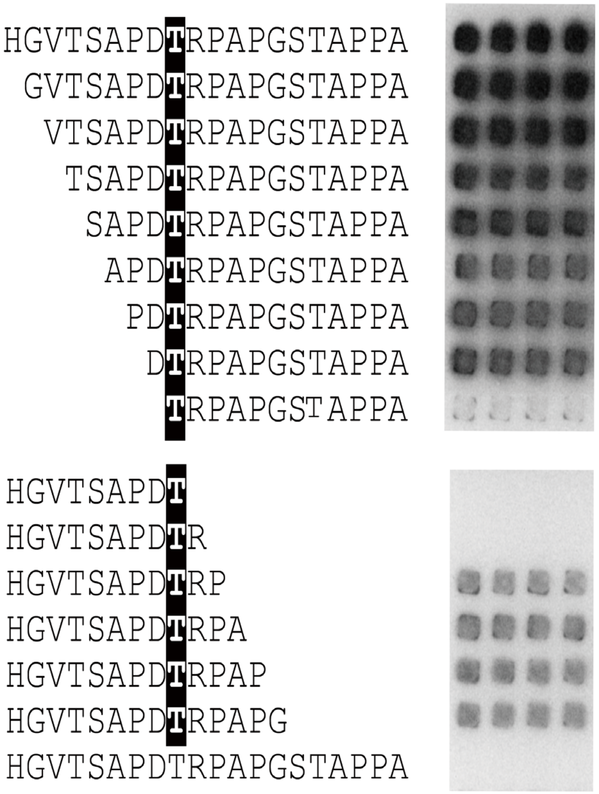

Fig. S1 | Antibody preparation and purification. **a**. A representative chromatograph of the affinity chromatographic purification of SN-131 eluted by using solution B (0.1 M glycine-HCl, pH2.7). **b**. Epitope mapping analyses of SN-131 antibody isolated by the affinity chromatography.

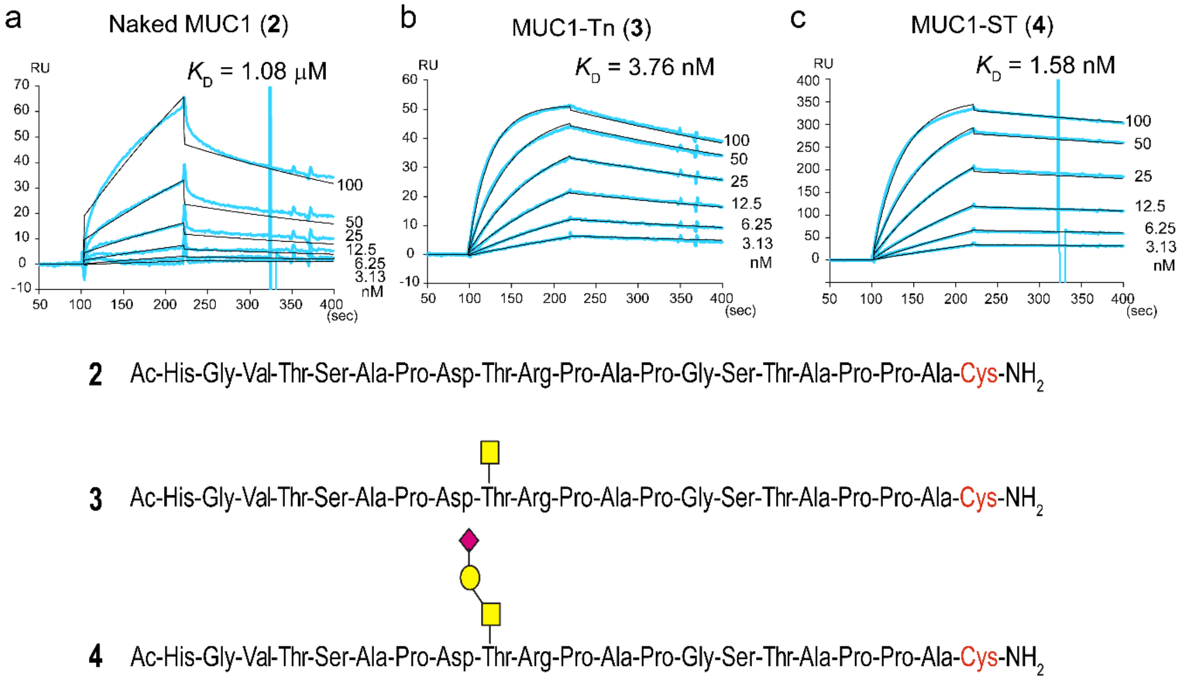

Fig. S2 | Affinity of antibody SN-131 Fab to the MUC1 TRD. a~c. Surface plasmon resonance curves for the binding of SN-131 Fab to the synthetic MUC1 TRD models **2**, **3**, and **4**, respectively. MUC1 TRD models having *C*-terminal cysteine residue were immobilized on a CM5 chip (GE Healthcare) through the maleimide coupling for the measurement of association of different concentrations of SN-131 Fab.

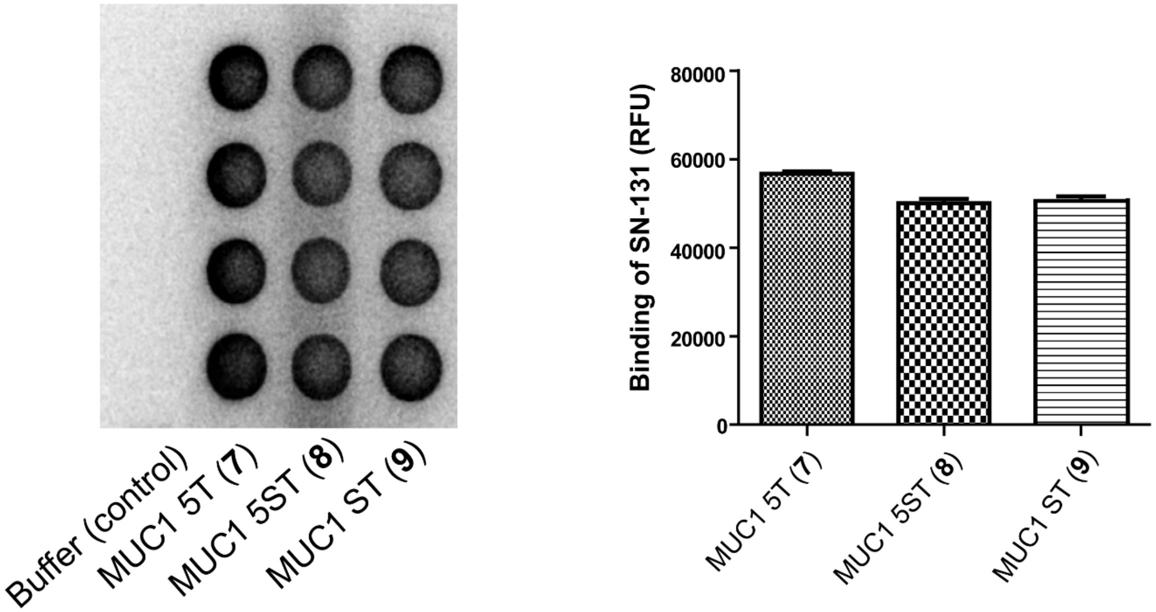

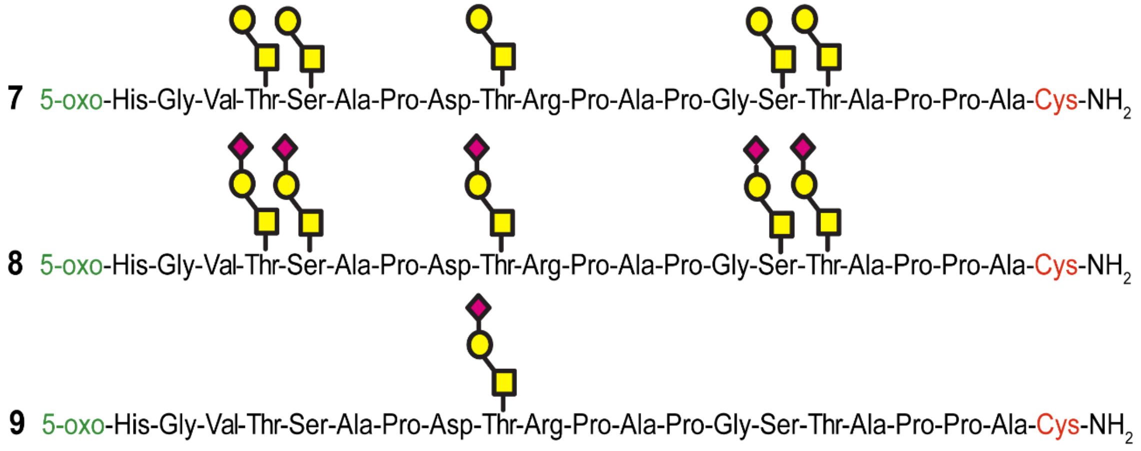

Fig. S3 | SN-131 binds dynamic epitopes in the MUC1 TRD independent of the modification at four other potential *O*-glycosylation sites. Microarray displaying bifunctional MUC1 5T (**7**), MUC1 5ST (**8**), and MUC1 ST (**9**) were employed for the immobilization by the oxime bond formation with AO-/PC-copolymer-coated surface for the characterization of the interaction of SN-131 (1 μg/mL) with MUC1 TRD, particularly the effect of occupation status at four other *O*-glycosylation sites on the binding to the dynamic epitope. Fluorescence due to the bound SN-131 probed by Cy3-labeled anti-mouse IgG polyclonal antibody for each 4 spots (left) was analysed quantitatively and represented as bar graph (right), respectively.

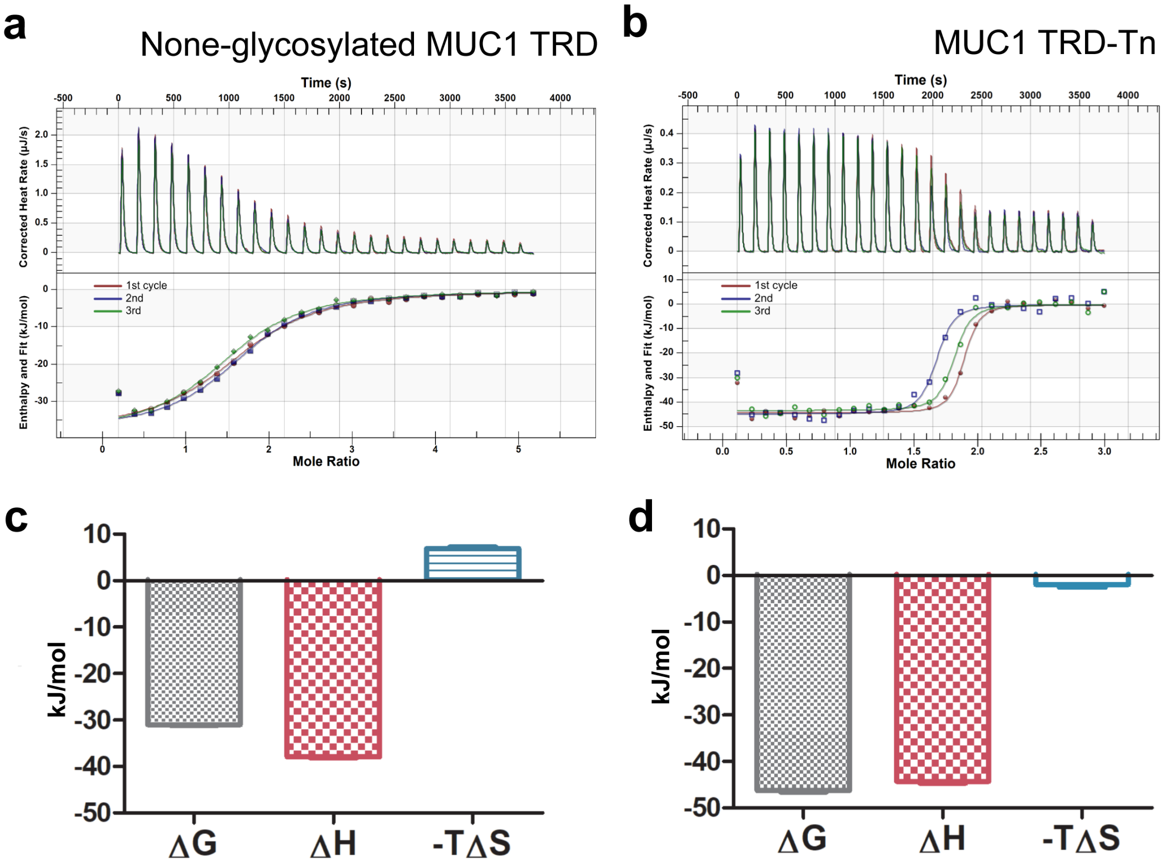

e

	Kd (M)	n	ΔG (kJ/mol)	ΔH (kJ/mol)	-TΔS(kJ/mol)
MUC1	3.70×10^{-6}	1.62	- 31.02	- 37.94	6.93
MUC1-Tn	7.85×10^{-9}	1.74	- 46.3	- 44.30	- 2.01

Fig. S4 | Thermodynamic significance of the glycosylation at an immunodominant Pro-Asp-Thr-Arg motif within MUC1 TRD in the SN-131 antibody-ligand interaction. Isothermal calorimetric titration (ITC) analyses for the interaction of SN-131 antibody with non-glycosylated MUC1 TRD (**5**) (**a**) and MUC1 TRD-Tn (**6**) (**b**), showing the binding isotherm. The experiments were performed three times, respectively. **c** and **d**. The changes of the thermodynamic parameters in the antibody-ligand (MUC1 or MUC1-Tn) binding, respectively. **e**. Table summaries thermodynamic binding parameters.

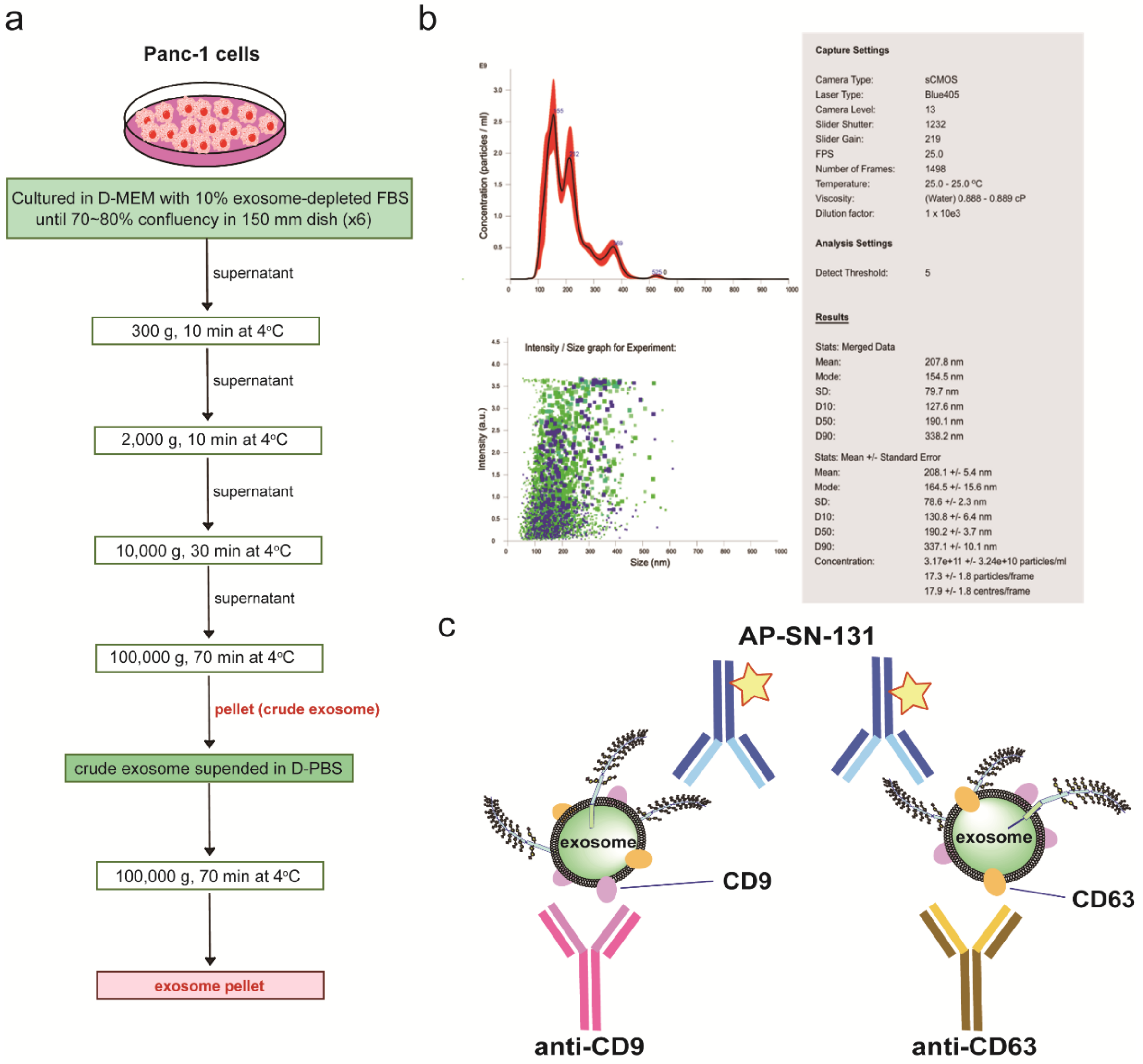

Fig. S5 | Exosomes released from Panc-1 cells display MUC1 TRD containing "dynamic epitope" recognized by SN-131. **a**. Isolation of exosomes (extracellular vesicles) from cultured Panc-1 cells was performed basically by ultracentrifugation. **b**. Exosome size and particle numbers were analysed using nanoparticle characterization system (Nano sight) equipped with a blue laser (405 nm). **c**. Schematic of a sandwich ELISA to demonstrate presence of MUC1 TRD bearing dynamic epitope of SN-131 on the exosomes captured by anti-CD9 and anti-CD63, antibodies interact with exosome makers CD9 and CD63, respectively. AP (alkaline phosphatase) labeled SN-131 with substrate (CDP-Star AP Substrate) were used for the quantitation of exosomal MUC1 TRD-STs.

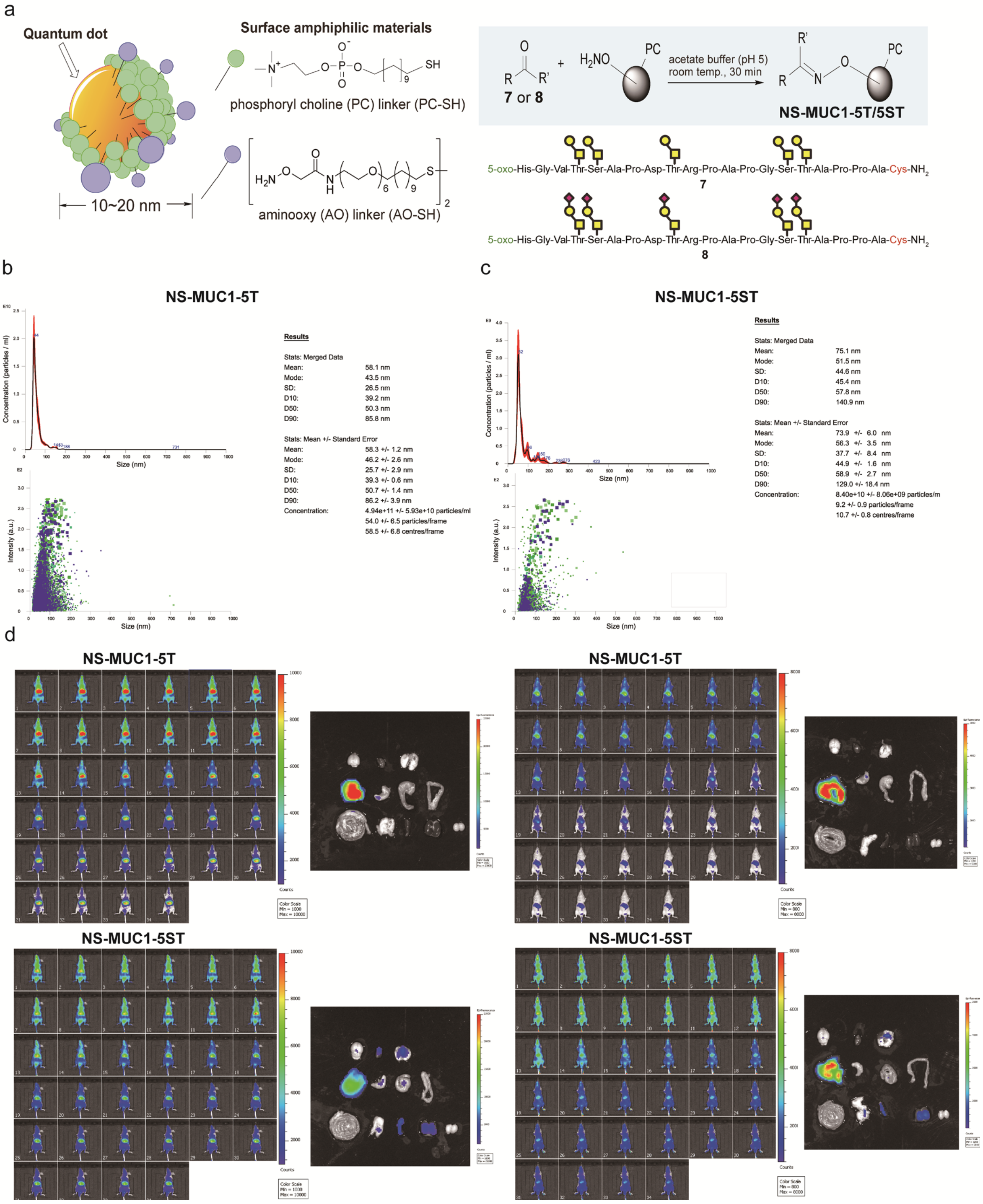
a
Quantum dot
Surface amphiphilic materials
phosphoryl choline (PC) linker (PC-SH)
aminooxy (AO) linker (AO-SH)
10~20 nm
acetate buffer (pH 5)
room temp., 30 min
7 or 8
NS-MUC1-5T/5ST
5-oxo-His-Gly-Val-Thr-Ser-Ala-Pro-Asp-Thr-Arg-Pro-Ala-Pro-Gly-Ser-Thr-Ala-Pro-Pro-Ala-Cys-NH2
7
5-oxo-His-Gly-Val-Thr-Ser-Ala-Pro-Asp-Thr-Arg-Pro-Ala-Pro-Gly-Ser-Thr-Ala-Pro-Pro-Ala-Cys-NH2
8
b
NS-MUC1-5T
Concentration (particles / ml)
Size (nm)
Intensity (a.u.)
Results
Stats: Merged Data
Mean: 58.1 nm
Mode: 43.5 nm
SD: 26.5 nm
D10: 39.2 nm
D50: 50.3 nm
D90: 85.8 nm
Stats: Mean +/- Standard Error
Mean: 58.3 +/- 1.2 nm
Mode: 46.2 +/- 2.6 nm
SD: 25.7 +/- 2.9 nm
D10: 39.3 +/- 0.6 nm
D50: 50.7 +/- 1.4 nm
D90: 86.2 +/- 3.9 nm
Concentration: 4.94e+11 +/- 5.93e+10 particles/ml
54.0 +/- 6.5 particles/frame
58.5 +/- 6.8 centres/frame
c
NS-MUC1-5ST
Concentration (particles / ml)
Size (nm)
Intensity (a.u.)
Results
Stats: Merged Data
Mean: 75.1 nm
Mode: 51.5 nm
SD: 44.6 nm
D10: 45.4 nm
D50: 57.8 nm
D90: 140.9 nm
Stats: Mean +/- Standard Error
Mean: 73.9 +/- 6.0 nm
Mode: 56.3 +/- 3.5 nm
SD: 37.7 +/- 8.4 nm
D10: 44.9 +/- 1.6 nm
D50: 58.9 +/- 2.7 nm
D90: 129.0 +/- 18.4 nm
Concentration: 8.40e+10 +/- 8.06e+09 particles/m
9.2 +/- 0.9 particles/frame
10.7 +/- 0.8 centres/frame
d
NS-MUC1-5T
NS-MUC1-5T
NS-MUC1-5ST
NS-MUC1-5ST

Fig. S6 | Exosome models carrying MUC1-5T and MUC1-5ST. **a**. Conjugation of synthetic MUC1 glycopeptide derivatives **7** and **8** with nanosome (NS) was performed according to the method reported previously.[31,32] NSs displaying mixed self-assembled monolayer of AO/PC linkers (10~20 nm) were conjugated with MUC1 glycopeptide derivatives **7** and **8** through oxime bond formation between ketone group and aminooxy group. **b**, **c**. Size and particle numbers of NS-MUC1-5T (left) and NS-MUC1-5ST (right) were analysed using nanoparticle characterization system (Nano sight) equipped with a blue laser (405 nm). **d**. Two independent results of the live animal NIR imaging for NSs carrying MUC1-5T and MUC1-5ST, indicating good reproducibility in these experiments.

Additional Data

HPLC profiles and MALDI-TOFMS of new compounds **1**, **2**, **4**, **7**, **8**, and **9**.

a

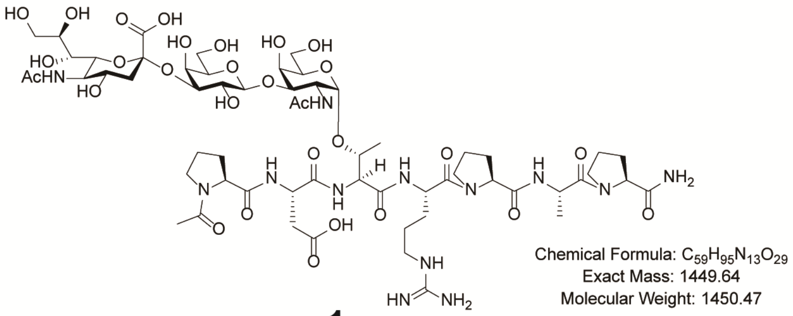

1

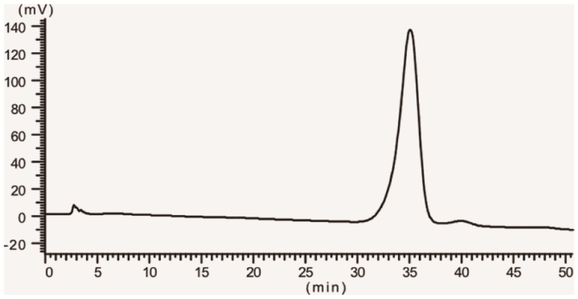

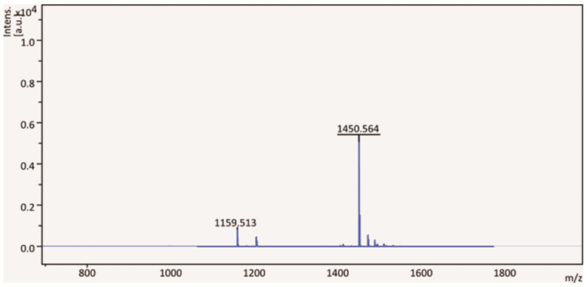

Analytical HPLC

Solvent A: 25 mM NH_4OAc/H_2O

Solvent B: 10% Solvent A in acetonitrile

Linier gradient of 2%~8% solvent B for 50 min.

Flow rate: 1 mL/min.

MALDI-TOFMS

Matrix: DHB, positive mode

Theoretical: 1449.64, Observed: $[M+H]^+$1450.564

b

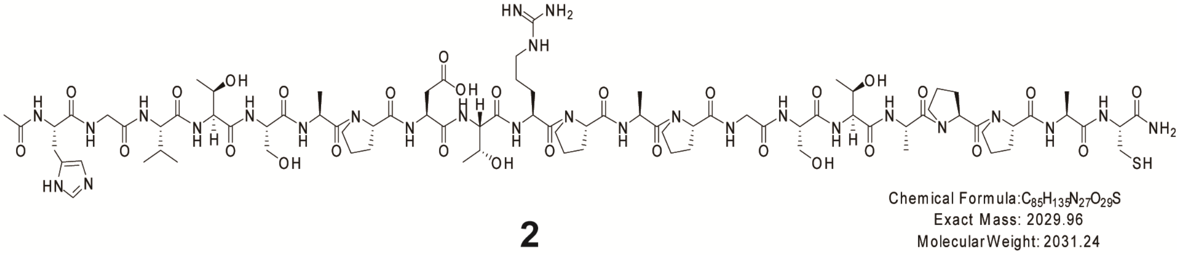

Chemical Formula: $C_{85}H_{135}N_{27}O_{29}S$
Exact Mass: 2029.96
Molecular Weight: 2031.24

2

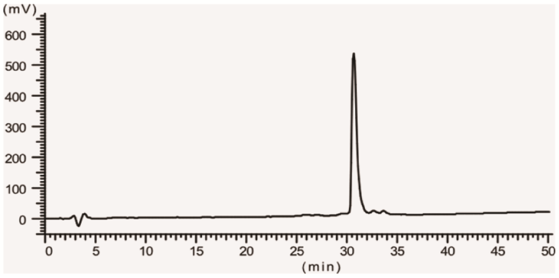

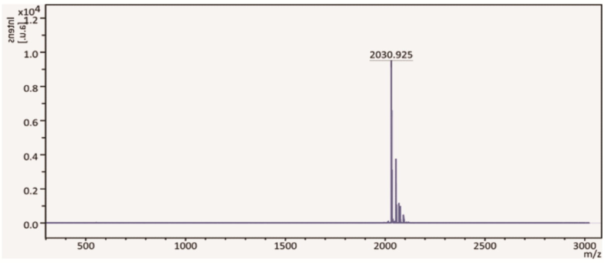

Analytical HPLC

Solvent A: 0.1% TFA in H_2O

Solvent B: 0.1% TFA in acetonitrile

Linier gradient of 5%~25% solvent B for 50 min.

Flow rate: 1 mL/min.

MALDI-TOFMS

Matrix: DHB, positive mode

Theoretical: 2029.96, Observed: $[M+H]^+$ 2030.925

C

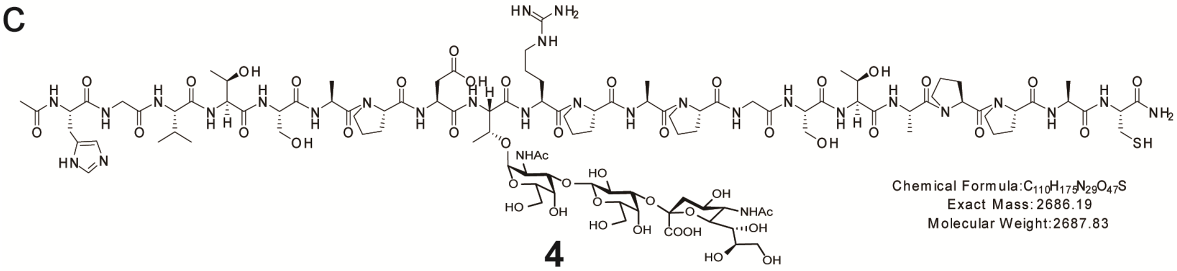

4

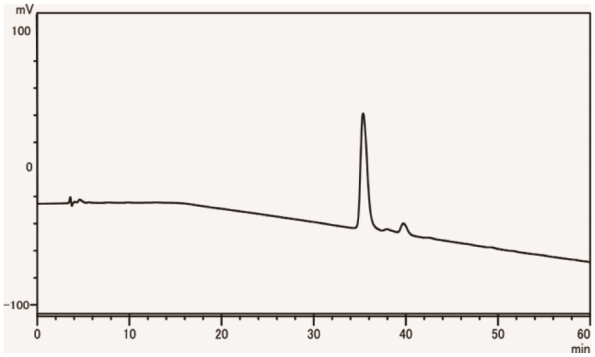

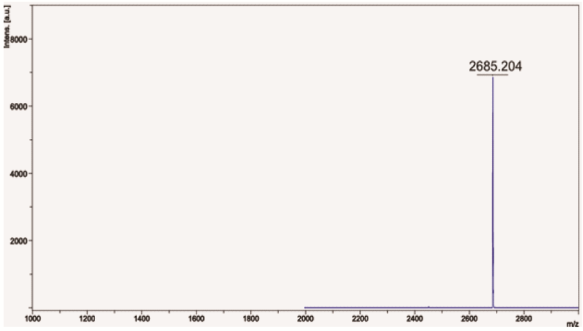

Analytical HPLC

Solvent A: 25 mM NH_4OAc/H_2O

Solvent B: 10% Solvent A in acetonitrile

Linier gradient of 2%~8% solvent B for 50 min.

Flow rate: 0.8 mL/min.

MALDI-TOFMS

Matrix: DHB, negative mode

Theoretical: 2686.19, Observed: $[M-H]^-$2865.204

d

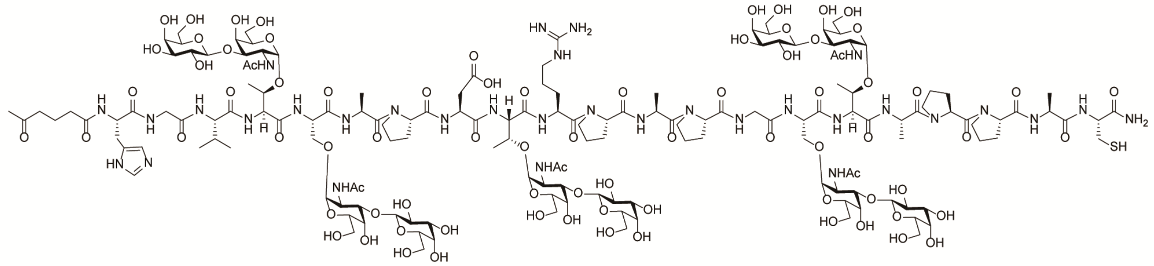

7

Chemical Formula: $C_{159}H_{256}N_{32}O_{80}S$
Exact Mass: 3925.67
Molecular Weight: 3928.00

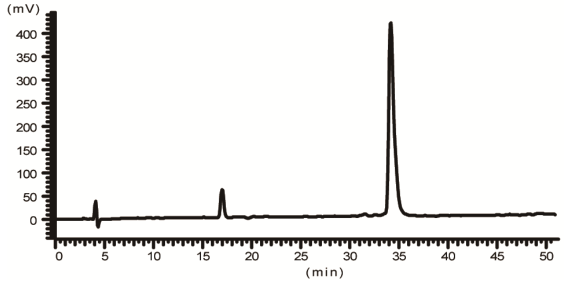

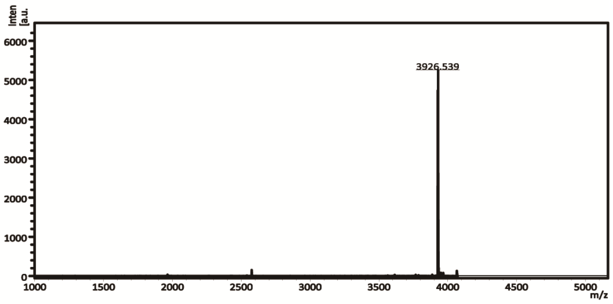

Analytical HPLC

Solvent A: 0.1% TFA in H_2O

Solvent B: 0.1% TFA in acetonitrile

Linier gradient of 5%~25% solvent B for 50 min.

Flow rate: 1 mL/min.

MALDI-TOFMS

Matrix: DHB, positive mode

Theoretical: 3925.67, Observed: $[M+H]^+$ 3926.539

e

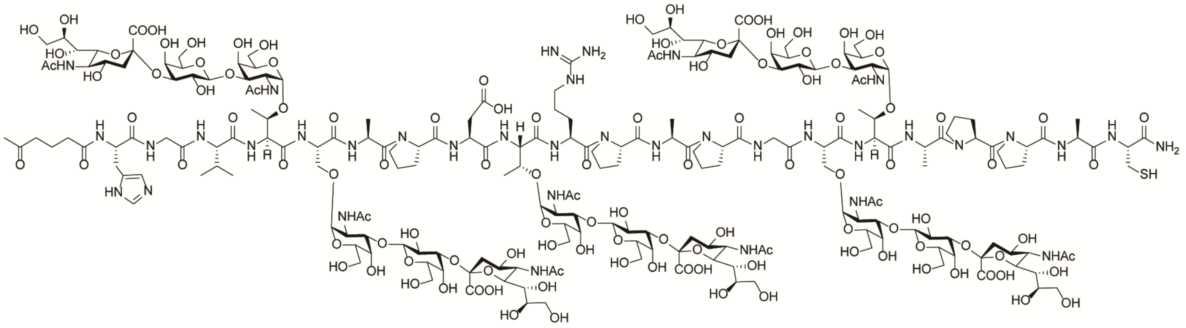

Chemical Formula: $C_{214}H_{341}N_{37}O_{120}S$
Exact Mass: 5381.14
Molecular Weight: 5384.28

8

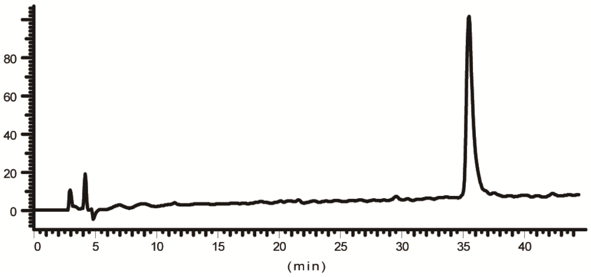

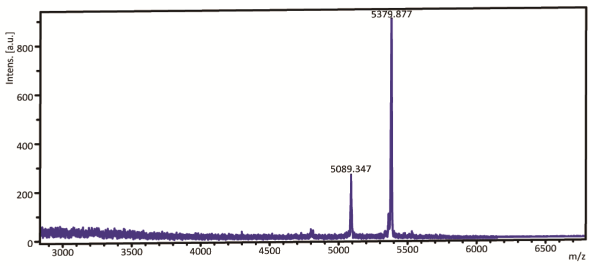

Analytical HPLC

Solvent A: 0.1% TFA in H_2O

Solvent B: 0.1% TFA in acetonitrile

Linier gradient of 1%~15% solvent B for 50 min.

Flow rate: 1 mL/min.

MALDI-TOFMS

Matrix: DHB, negative mode

Theoretical: 5381.14, Observed: $[M-H]^-$ 5379.877

f

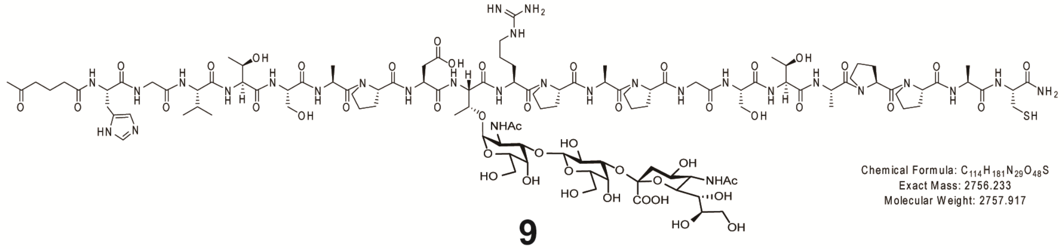

9

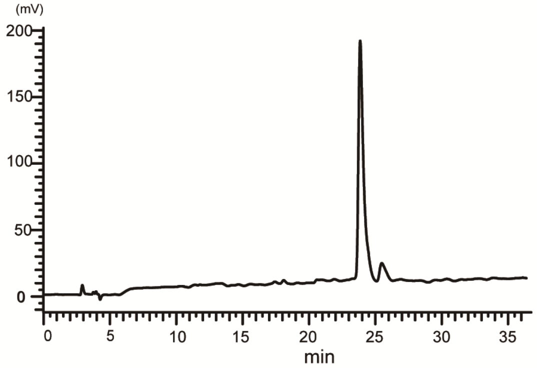

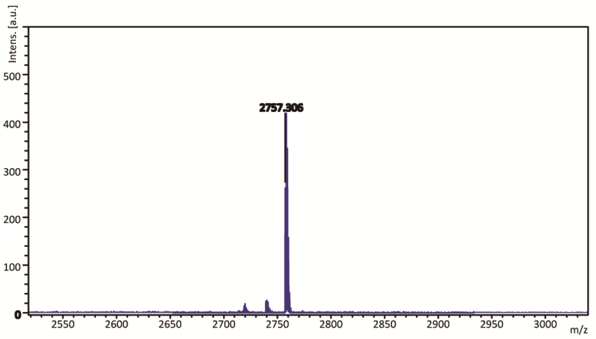

Analytical HPLC

Solvent A: 0.1% TFA in H_2O

Solvent B: 0.1% TFA in acetonitrile

Linier gradient of 1%~15% solvent B for 50 min.

Flow rate: 1 mL/min.

MALDI-TOFMS

Matrix: DHB, positive mode

Theoretical: 2756.233, Observed: $[M+H]^+$ 2757.306
